# Unravelling phenotypic variations and establishing a core collection in mungbean for accelerating the crop improvement programs

**DOI:** 10.3389/fpls.2026.1743562

**Published:** 2026-02-25

**Authors:** Rajwant K. Kalia, Gayacharan, Deepak Khanderao Patil, Dhammaprakash Pandhari Wankhede, J. Aravind, Neeta Singh, Prakash Kumar, Amit Kumar Singh, Pooja Panchariya, Manoj Choudhary, Kantilal Solanki, Rajesh K. Kakani, Reena Rani, H. R. Mahla, Sunil Gomashe, M. Latha, Neelam Shekhawat, Rakesh Pathak, Kuldeep Singh, Gyanendra Pratap Singh

**Affiliations:** 1 ICAR-Central Arid Zone Research Institute, Jodhpur, Rajasthan, India; 2 ICAR-National Bureau of Plant Genetic Resources, New Delhi, Delhi, India; 3 Agricultural Research Station, Badnapur, VNMKV, Parbhani, Maharashtra, India; 4 ICAR-Indian Agricultural Statistics Research Institute, New Delhi, Delhi, India; 5 ICAR-National Bureau of Plant Genetic Resources, RS, Akola, Maharashtra, India; 6 ICAR- National Bureau of Plant Genetic Resources, RS, Thrissur, Kerala, India; 7 ICAR- National Bureau of Plant Genetic Resources, RS, Jodhpur, Rajasthan, India; 8 International Crop Research Institute for Semi-arid Tropics, Patancheru, Hyderabad, India

**Keywords:** agro-morphological variation, breeding values, core development, diversity analysis, genetic phenotypic variability, greengram

## Abstract

Mungbean [*Vigna radiata* (L.) R. Wilczek], is an Indian-origin legume crop and is emerging as an excellent food for human health and nutrition globally. However, the narrow genetic base in its released varieties is a bottleneck in mungbean yield enhancement. Therefore, 3903 accessions of mungbean were characterized to identify trait-specific donors, understand potential traits for breeding purposes, and select a core collection for enhancing germplasm utilization in the mungbean breeding program. A common set of 28 phenotypic traits was used for evaluation at two locations for two years (for 2019 and 2020) in an augmented block design. A substantial amount of phenotypic variation was observed in mungbean collections as revealed by diversity indices, frequency distribution, hierarchical clustering, and principal component analysis. Traits such as plant biomass, days to 50% flowering, days to maturity, plant height, and number of seeds/pod showed higher levels of heritability and genetic gains. The core development strategy involved the development of five independent core sets of 400 accessions based on different statistical methods. Among the five sets, a core collection i.e., EN100 (average entry-to-nearest-entry distance with 100% weightage), was identified as the best among all five core sets based on quality evaluation indices. The chosen core collection was further compared with the entire collection using various statistical parameters. The core collections showed optimum results based on the various quality assessment parameters such as mean difference (MD, 66.67%), variance difference (VD, 100%), coincidence rate of range (CR, 97.4%), variable rate of range (VR, 119.6%), class coverage (100%) and a higher level of coefficient of variation (CV) for all the quantitative traits. These findings provide insights into the mungbean germplasm diversity, which will play a significant role in the mungbean improvement programs. Additionally, the diversity-rich core collection will be a ready resource for breeders and researchers to address various research problems.

## 1 Introduction

Mungbean (*Vigna radiata* L. Wilczek), also popularly known as greengram, is an important food legume crop of southern and southern East Asia. Archaeological evidence and the distribution of the progenitor species (*V. radiata* var. *sublobata* (Roxb)) of mungbean indicate the Neolithic domestication of mungbean on the southern peninsula of India, more precisely, north of the Krishna River (Zukovsky, 1962; Fuller, 2011; Ambika et al., 2022). Mungbean crops have spread to Southeast Asia and East and Central Asia, including Western China, Mongolia, Afghanistan, Iran and Russia (Kang et al., 2014; Ileana, 2023). Mungbean cultivation has sustained civilizations for the past 4000–6000 years (Kang et al., 2014). Mungbean plants have evolved with a myriad of adaptation mechanisms, which make it a suitable crop for tropical and subtropical climatic conditions (Bhardwaj et al., 2023). The evolution of mungbean crops is not solely driven by human-led domestication; rather, climatic conditions have played a great role throughout the journey of mungbean (Ileana, 2023). Therefore, mungbean plants, which have a short life cycle, have become more relevant crops with the possibility of further spreading their cultivation to non-traditional areas amid climate change scenarios.

The annual global production of mungbean is approximately 6 million tons (MT), with an average yield of approximately 720 kg/ha (Nair and Schreinemachers, 2020; Gayacharan et al., 2023). India is a major mungbean producer, with 41% (2.45 MT) of global mungbean production. Mungbean is also considered one of the most significant grain legumes because of its nutrient-rich composition. It provides a range of essential nutrients, such as protein (21-31%), dietary fiber (ca. 17%), vitamins, and minerals (ca. 3.5%), making it an excellent plant-based nutrient source for vegetarians and vegans. Mungbean plants are excellent sources of minerals such as magnesium (mg), phosphorus (P), potassium (K), and vitamins such as thiamine (vit. B1) and riboflavin (vit. B2) (Kanishka et al., 2023). Mungbean seeds are also low in fat (1.3%) and have a low glycemic index, making them a healthy source of a balanced diet.

Despite the immense role of mungbean crops in economic, ecological, nutritional, and food security, crop yields have remained poor for the last several decades. The major cause of poor yield enhancement in mungbean plants is poor utilization of mungbean germplasm in breeding programs. In several instances, the utilization of crop variability available in genebanks or *in situ on-farms* is the key to the success of a breeding program. Over 43,000 accessions of mungbean are conserved in genebanks worldwide, and more than 9,000 accessions of mungbean are kept in black box systems at the Svalbard Global Seed Vault as safety duplicates (Nair et al., 2013a; Gayacharan et al., 2020a). However, identifying and selecting the desired variability in larger collections has become a challenging task for researchers. To overcome this hindrance, the “core collection” concept was devised (Frankel, 1984). In this direction, a mungbean core collection of 1,481 accessions was developed using the collections conserved at the genebank of the AVRDC-The World Vegetable Centre, Taiwan (Schafleitner et al., 2015). The core collection was further genotyped using 20 SSR markers, and a mini-core collection of 289 accessions was developed (Schafleitner et al., 2015). The mini-core has been extensively utilized to identify donors and QTLs for various important traits (Sokolkova et al., 2020; Reddy et al., 2020; Breria et al., 2020a, 2020b; Aski et al., 2021), showing the importance of core collection in crop improvement programs. Although similar studies characterizing mungbean germplasm have been conducted to determine genetic diversity and develop core collections (Bisht et al., 1998; Gayacharan et al., 2020b), no other large-scale mungbean characterization has been done. India’s National Seed Genebank conserves more than 4000 accessions of mungbean, but the majority of the accessions were not characterized. Therefore, in this study, the entire mungbean collection conserved in India’s National Genebank, the majority of which are of Indian origin, was characterized using extensive phenotypic characteristics to improve mungbean germplasm utilization in crop improvement programs. Further, to enhance the mungbean germplasm utilization in the mungbean crop improvement program, a core collection with a sizeable number has been developed.

## 2 Materials and methods

### 2.1 Mungbean germplasm

**Figure 1:**
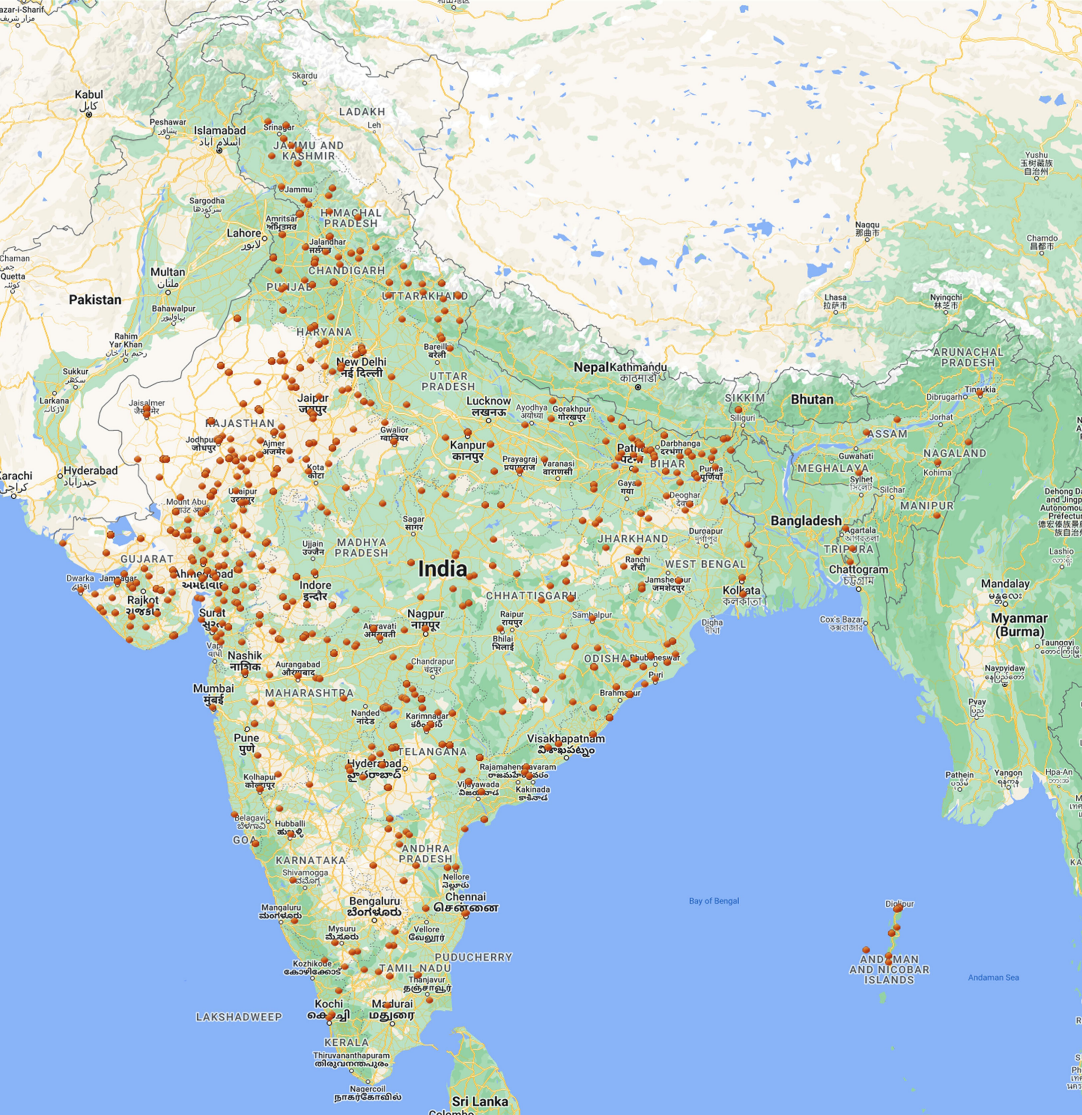
Distribution of indigenous mungbean germplasm. The figure indicates a good representation of the mungbean germplasm from all the mungbean crop growing areas. (Source: Database of ICAR-NBPGR Genebank http://pgrinformatics.nbpgr.ernet.in/akmu/Default.aspx).

A total of 4100 mungbean germplasm accessions were drawn from the Indian National Genebank, ICAR-National Bureau of Plant Genetic Resources, New Delhi. After removal of the duplicate accession (based on passport information), 3903 unique mungbean accessions were included in this study. Out of the 3903 accessions, 3411 accessions originated from various parts of India (**Figure 1**). Another 491 accessions originated from Taiwan (215 acc.), China (49 acc.), Thailand (40), Sri Lanka (10 acc.), Japan (9 acc.), USA (7 acc.), Hungry (3 acc.), Pakistan (92 acc.) and one accession each from Costa Rica, Madagascar, New Zealand, the Philippines, Russia, Suriname and Australia, while 149 exotic accessions were from unknown sources.

### 2.2 Experimental design and phenotyping method

Mungbean germplasm were grown at two locations, *viz*., Jodhpur, Rajasthan (26.25°N, 72.99°E), and Badnapur, Maharashtra (19.86°N, 75.70°E), which fall under distinct agro-climatic zones (**Supplementary Table S1**). Jodhpur, Rajasthan, has a hot, arid climate characterized by extreme temperatures and low rainfall. While Badnapur, Maharashtra, has a tropical wet and dry climate, with relatively higher rainfall and a cooler monsoon season. The soil types of both locations were distinct. The Badnapur location soil type was clay-loam of vertisol order, having a field capacity of 48%, while the Jodhpur soil type was loamy-sand with a field capacity of 18%. Sowing was done in an augmented block design (ABD-II) during the monsoon seasons of 2019 and 2020. A total of five checks, *viz*., Virat (IPM-205-7), Samrat (PDM-139), IPM2-14, SML-668 and MH-421, were repeated twice for each of the 50 blocks. The data was recorded for 17 quantitative traits viz., 100-seed weight, days to 50% flowering, days to 80% maturity, number of pods/cluster, number of pods/plant, number of primary branches/plant, peduncle length, petiole length, plant biomass, plant height, pod length, pod weight/plant, stem diameter, terminal leaf length, terminal leaf width, grain yield/plant, and number of seeds/pod. Three data points were recorded for each of these quantitative traits. Seed morphological parameters such as the seed area, seed length, seed breadth and seed roundness were recorded based on image analysis of 5 seeds per accession. Seven qualitative traits viz., hypocotyl color, seedling vigor, growth habit, raceme position, pod attachment to peduncle, pod pubescence and seed color were also recorded as per the characterization descriptors for mungbean (IBPGR, 1980).

### 2.3 Statistical analyses used to sample the core collection

The average values for all the quantitative traits were used for the analyses. Data transformation was not required after careful checking of the residuals. The adjusted mean values and Analysis of Variances (ANOVA) for all the environments for ABD were estimated in R with the package ‘augmented RCBD’ (Aravind et al., 2019). The Bartlett’s χ^2^ test was used to assess the homogeneity of error variances among multi-environment data (**Supplementary Table S2**). The frequency distribution of qualitative and quantitative traits of the data from both locations was also performed to visualize the environmental influence on the traits’ expression (**Supplementary Figures S2**–**S4**). These analyses indicated a significant level (P < 0.005) of environmental influence on phenotypic expression of all the quantitative traits in mungbean germplasm across the locations. Therefore, a single season of data from Jodhpur, Rajasthan, for the year 2020, was selected for further analyses to designate a set of accessions as the core collection (CC). The monsoon-season climate of Jodhpur is highly conducive to mungbean phenotypic expression and is broadly representative of agro-climatic conditions across Rajasthan and other northern Indian states. Therefore, the findings of this study are expected to have broader applicability and relevance for mungbean improvement programs in the region.

The quantitative data on 21 agromorphological traits were used to extract a representative set of diverse accessions (core collection), with a threshold of 10% set for selecting the number of accessions in the diverse panel of the entire collection. Further, to enhance the useful variability and geographic representation in the core set, biased selections were preprogrammed in the CoreHunter program. Biased selections were performed for promising accessions of economic traits, unique and rare traits, and accessions contributing a higher level of variance to the major principal components (PCs). At least one accession was assumed to represent each geographical location in the final core set. Following this strategy, a core collection of 400 mungbean accessions was developed (**Supplementary Table S1**).

Initially, a total of five types of core sets were assembled using different methods viz., PowerCore (a), PCSS, principal component score strategy (b), average entry-to-nearest-entry distance with 100% weightage (EN100) (c), average accession-to-nearest-entry distance with 100% weightage (AN100) (d), and combined EN and AN with equal weightage (EN50:AN50) (e). The last three methods i.e., (c), (d) and (e) are of the CoreHunter (version 3.2.1) program. The PowerCore program employs an advanced M strategy, combined with a modified heuristic search algorithm, to maximize allelic richness and minimize redundancy in the core set (Kim et al., 2007). Whereas, PCSS employs principal component analysis to eliminate collinearity among variables and identify diverse accessions based on their relative contribution (Noirot et al., 1996). The EN method is used to maximize the diversity whereas, algorithm involved in the AN method enhances the representativeness of the diversity level in the derived core collections (Odong et al., 2013; De Beukelaer et al., 2018).

### 2.4 Comparative evaluation of four core sets for designating the final core collection

All four core sets were statistically evaluated to identify the best core set that best represents the genetic diversity present in the entire collection of mungbean used in this study. For this purpose, statistical parameters such as summary statistics, genetic distances as described by Odong et al. (2013), mean difference percentage (MD%), variance difference percentage (VD%), coincidence rate of range (CR%), and variable rate of coefficient of variance (VR%) were estimated for quantitative traits as described by Hu et al. (2000). The consistency in difference among entire collection and selected core set for mean and variances was done using Sign test as described by Basigalup et al. (1995) and Tai and Miller (2001). The Mantel test was performed to check the significance of correlation between the correlation matrices of entire collections and selected core set (Mantel, 1967). For qualitative traits, Shannon’s diversity index (Shannon, 1948) was used to measure the diversity of the entire population as well as the core collection. Shannon’s diversity index (H’) takes into account the relative abundance of descriptor states within the population. The higher the H’ value is, the greater the diversity within the population. The Shannon’s evenness index (J’), also known as Shannon’s evenness index or the Shannon–Wiener evenness index, is a measure of the evenness of species (descriptor state in this study) distribution within a population. J’ describes how equally each descriptor state of a trait is distributed among the individuals of the population. H_max_ is the log value of the number of descriptor states. H_max_ indicates the maximum potential diversity within a population.

After selection of the core set showing maximum diversity and representativeness of the entire mungbean germplasm diversity, it was subjected to comparative evaluation with the entire set of collections for quantitative traits using the R package EvaluateCore (Aravind et al., 2020). The Newman-Keuls test (Newman, 1939; Keuls, 1952), a *post-hoc* test and t-test, were used to compare means. The Levene’s test (Levene, 1960) was used to test the homogeneity of variance. The Wilcoxon rank test (Wilcoxon, 1945), a non-parametric test, was used for comparison of frequency distribution. The frequency distribution using box-plots, bar charts and area charts was done to visualize the patterns and trends in the datasets. The quantile-quantile (Q-Q) plots (Wilk and Gnanadesikan, 1968), along with other tests such as Kullback-Leibler distance (Kullback and Leibler, 1951), Kolmogorov-Smirnov (Massey, 1951), and Anderson-Darling goodness of fit test (Anderson and Darling, 1954) were drawn to visualize the differences in distribution between the core collection and entire collections. The principal component analysis (PCA), a method to visualize the dataset in a low-dimensional scale, was performed to compare the amount of explained variance, the direction of variance in major principal components (PCs), and the relationship among variables and observations. The correlation among quantitative traits was also estimated using Pearson’s coefficient (Pearson, 1895), and a correlogram matrix was drawn (Friendly, 2002).

### 2.5 Estimation of genetic variability parameters

Analysis of variance (ANOVA) for augmented block design (ABD) for each quantitative trait on the entire collection was done using R version 4.0.4 and SAS 9.4 software. To understand the usefulness of the quantitative traits in the crop improvement program, the genetic variability parameters such as phenotypic variance (Vp), genotypic variance (Vg) were calculated as explained by Bhagasara et al. (2017). These variances were further used in the estimation of genotypic coefficient of variation (GCV) and phenotypic coefficient of variation (PCV) (Burton, 1952), and broad sense heritability (h^2^) (Lush, 1940). Further classification of h^2^ into low (<30%), medium (30-60%), and high (>60%) was followed from Robinson (1966). The genetic advance (GA) and genetic gain (GG) were calculated as explained by Johnson et al. (1955).

## 3 Results

### 3.1 Phenotypic variation in mungbean collections

**Figure 2:**
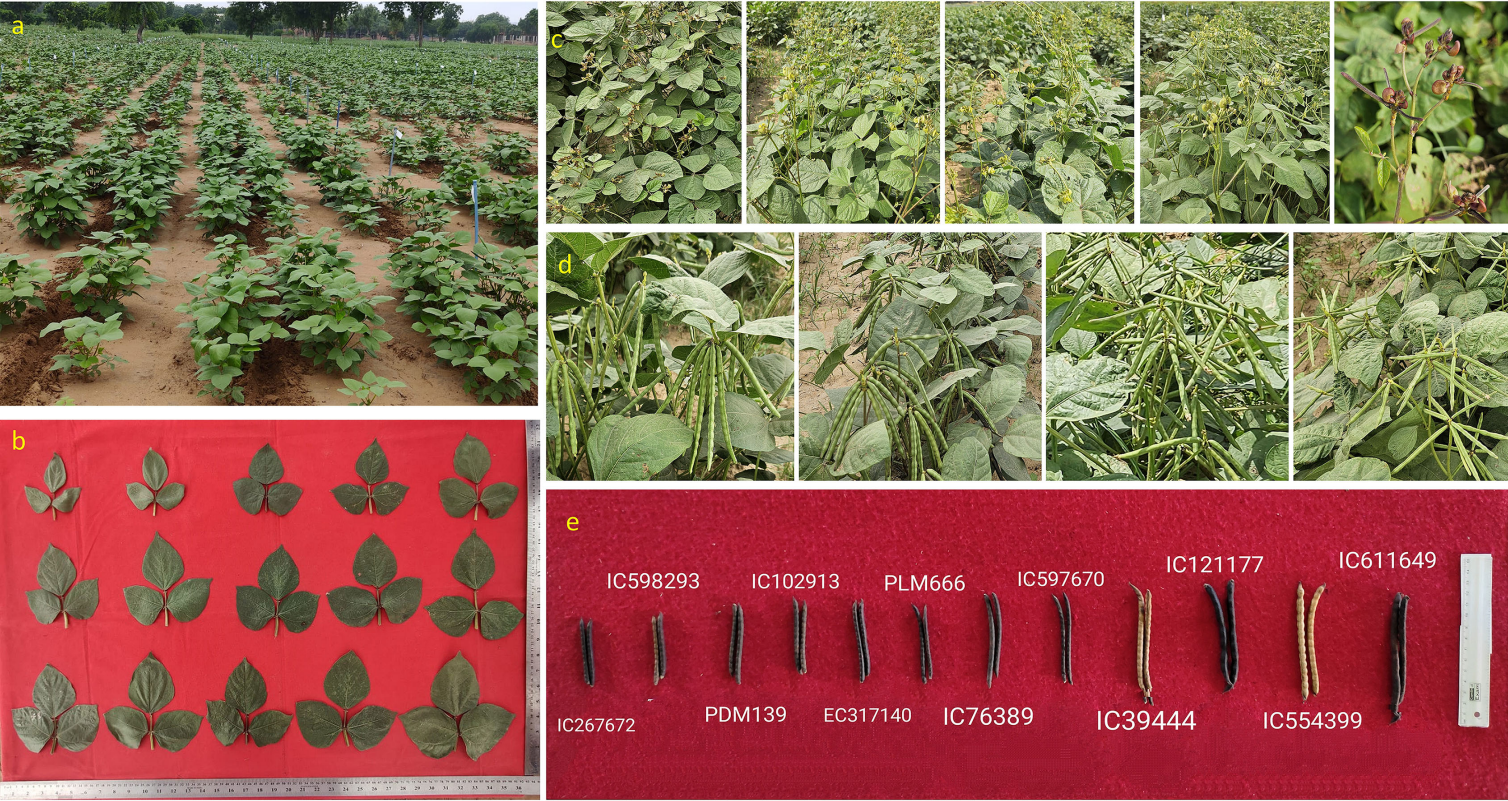
Representation of phenotypic variability in mungbean: partial field view of the experiment in pre-flowering stage **(a)**, variation in size of 4^th^ leaf in mungbean germplasm **(b)**, variation in inflorescence color, growth habit **(c)**, variability for pod orientation in peduncle **(d)**, and variability for dry pod length, width and color **(e)**.

**Figure 3:**
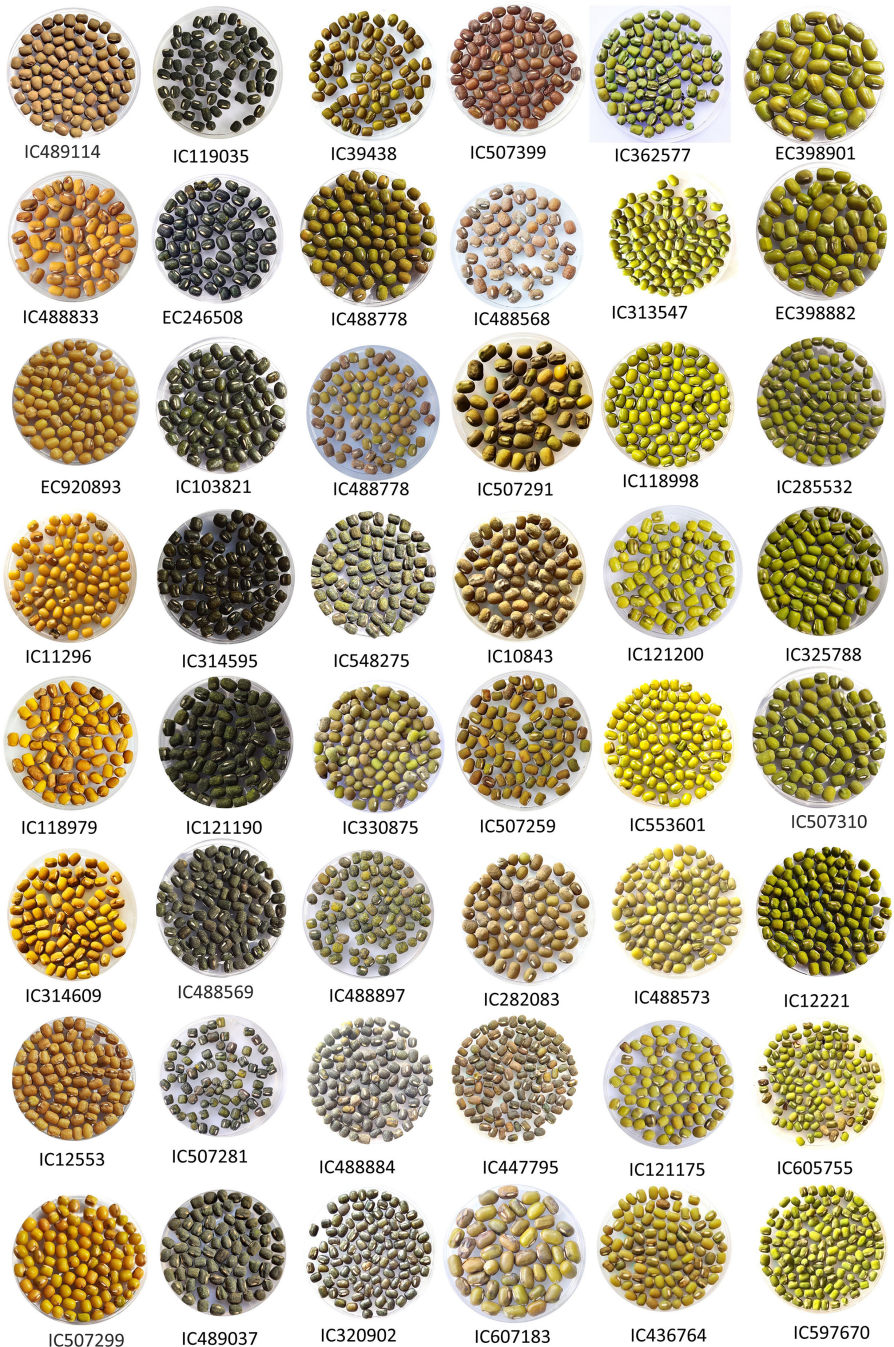
Highlights of seed morphological variability for seed coat color, seed size, seed surface luster, and seed shape of some of the representative accessions of mungbean germplasm used in this study.

**Figure 4:**
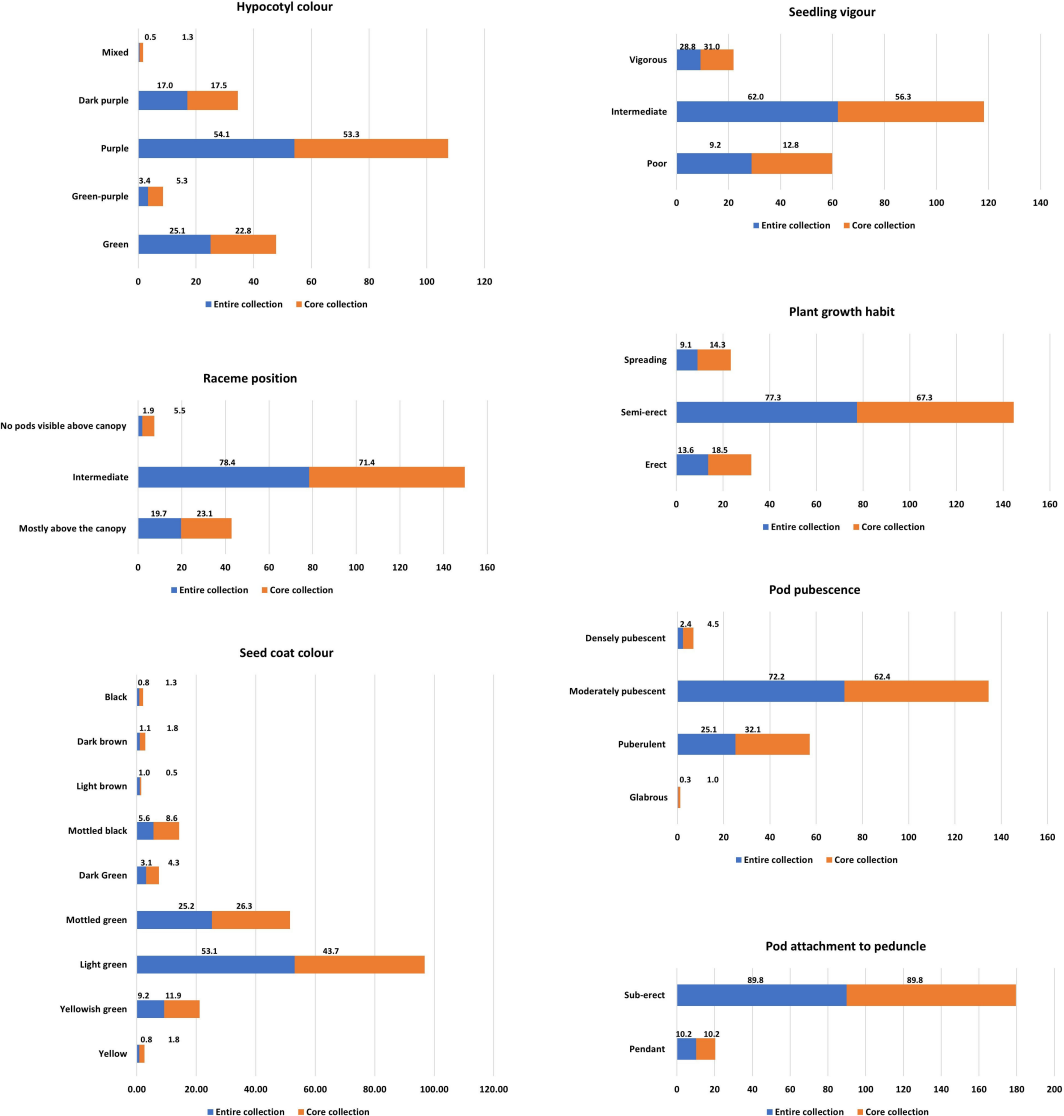
Frequency distribution of seven qualitative traits in the entire collection and core collection. Values on bars indicate the proportion of the descriptive state of the trait’s representation on the entire collections (3903 acc.) and core collections (400 acc.).

**Figure 5:**
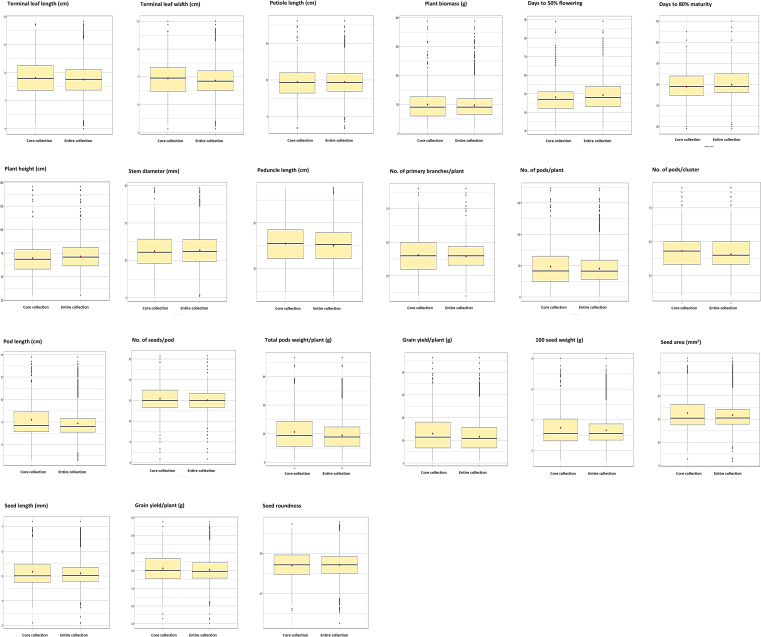
Boxplots highlighting the frequency distribution of 21 quantitative traits in the entire collections (EC) and core collections (CC).

**Figure 6:**
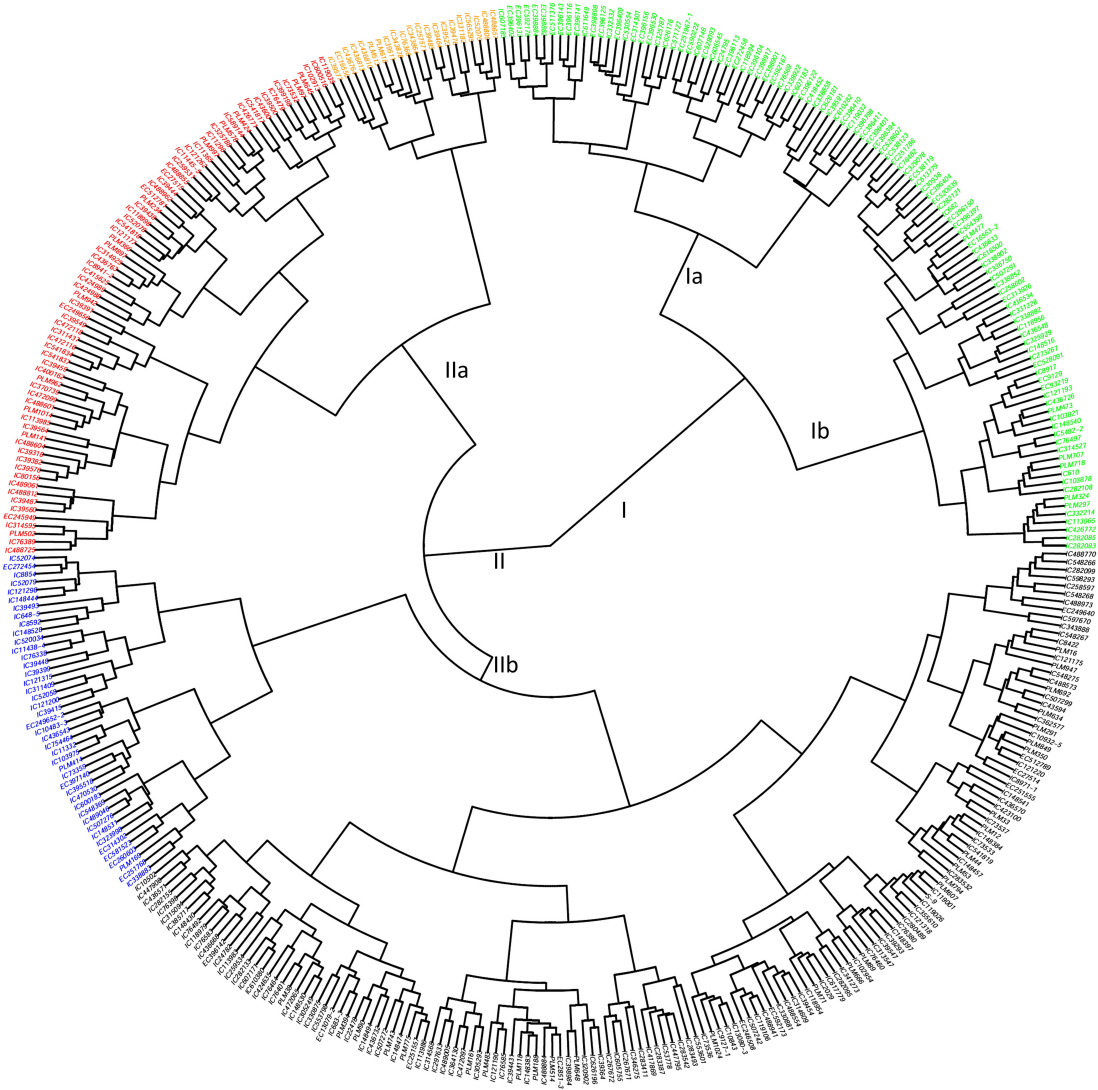
Circular hierarchical clustering of core collection based on quantitative traits’ data with respective accessions at the end of each bar. The core collections were grouped into two major clusters (I & II), and further, each cluster was divided into two sub-clusters, which are further sub-clustered. The accessions’ cluster and passport information are given in the supplementary information (**Supplementary Table S1**).

The characterization revealed a substantial amount of phenotypic variation in mungbean germplasm for various important traits, such as PB, PDL, NPB, NPPP, NPPC, PWPP, GY, SW (**Figures 2**, **3**; **Tables 1**, **2**). Higher level of GCV and PCV was observed for the traits viz., PDL, NPPC, PBP, NPPP, PBP, GY, SW, and PW (**Table 1**; **Supplementary Figure S5**). The h^2^ was also observed higher for traits such as PH, PB, DFF, DM, PL, NSPP, SW, SA, SL and SB (**Table 1**; **Supplementary Figure S6**). A similar trend was also observed for genetic gains (**Table 1**). Based on critical difference (CD at 5%), promising accessions for phenotypic traits were also identified (**Supplementary Table S4**). Other statistical analyses such as, analysis of variance (**Supplementary Table S3**), frequency distribution (**Figures 4**, **5**; **Supplementary Figures S2**, **S3**), PCA (**Table 3**; **Figure 6**; **Supplementary Figure S4**), hierarchical clustering (**Figure 6**), and diversity indices (**Table 3**), showed a significant amount of variation present in the mungbean collections. The frequency distribution of quantitative traits showed well-distributed diversity across the ranges (**Figure 4**; **Supplementary Figure S2**). The phenotypic variability for some of the important traits is highlighted in **Figures 2**, **3**.

**Table 1:** Estimation of genetic variability parameters for quantitative traits using the entire set of mungbean collections.

Trait	PCV	GCV	h^2^ (%)	h^2^ category	GA	GA category	GG	GG category
TLL	5.98	13.17	20.62	Low	0.48	Low	5.60	Low
TLW	8.16	16.03	25.92	Low	0.58	Low	8.57	Low
PH	17.18	19.74	75.70	High	22.02	High	30.83	High
PTL	11.62	19.35	36.05	Medium	1.40	Low	14.39	Medium
PDL	21.05	28.30	55.32	Medium	4.85	Low	32.30	High
PL	13.15	15.54	71.63	High	1.81	Low	22.96	High
NPPC	23.10	31.52	53.70	Medium	1.42	Low	34.92	High
SD	15.16	19.31	61.62	High	2.80	Low	24.55	High
NPB	20.42	28.97	49.68	Medium	1.18	Low	29.69	High
NSPP	5.96	8.58	48.36	Medium	1.03	Low	8.56	Low
NPPP	29.75	52.26	32.42	Medium	15.95	Medium	34.95	High
PB	38.09	45.61	69.76	High	31.69	High	65.63	High
DFF	16.48	17.84	85.40	High	15.48	Medium	31.42	High
DM	7.53	9.55	62.10	High	9.76	Low	12.23	Medium
GY	23.41	57.80	16.40	Low	2.29	Low	19.55	Medium
SW	25.83	30.03	74.01	High	1.52	Low	45.85	High
SA	16.66	18.97	77.14	High	4.79	Low	30.19	High
SL	8.65	9.93	75.80	High	0.79	Low	15.53	Medium
SB	7.28	8.98	65.74	High	0.49	Low	12.18	Medium
SR	2.30	4.19	30.03	Medium	0.02	Low	2.60	Low
PWPP	21.08	54.55	14.93	Low	3.19	Low	16.80	Medium

Vp, phenotypic variance; Vg, genotypic variance; PCV, phenotypic coefficient of variation; GCV, genotypic coefficient of variation; h2, broad sense heritability; GA, genetic advance; GG, Genetic gain; TLL, Terminal leaf length (cm); TLW, Terminal leaf width (cm); PTL, Petiole length (cm); PB, Plant biomass (g); DFF, Days to 50% flowering; DM, Days to maturity 80%; PH, Plant height (cm); SD, Stem diameter (mm); PDL, Peduncle length (cm); NPB, Number of primary branches, NPPP, Number of pods per plant; NPPC, Number of pods/cluster; PL, Pod length (cm); NSPP, Number of seeds/pod; PWPP, Pod weight/plant (g), GY, Grain yield/plant(g); SW, 100 seed weight. (g); SA, Seed area (mm^2^); SL, Seed length (mm); SB, Seed Breadth (mm); SR, Seed Roundness.

**Table 2:** Comparison of summary statistics, skewness, kurtosis, and other statistical parameters between the entire collection and the derived core set for various quantitative parameters.

Traits	Entire collection						Core collection (EN100)						Difference between the entire collection and the core EN100			
Traits	Range	Mean ± SD	CV % (P)	IQR	Skewness	Kurtosis	Range	Mean ± SD	CV % (P)	IQR	Skewness	Kurtosis	Newman-Keuls test^a^	t-test^b^	Levene’s test^c^	Wilcoxon rank test^d^
TLL	5.0-12.7	8.5 ± 1.1	13	1.47	0.11	0.2	5.3-12.5	8.6 ± 1.3	15.6	1.77	0.14	0.11	0.06^ns^	0.1^ns^	0.00**	0.10^ns^
TLW	3.3-11	6.7 ± 1.1	15.9	1.43	0.17	0.13	3.3-11	6.9 ± 1.3	19.2	1.7	0.27	0.11	0.01**	0.02*	0.00**	0.03*
PTL	3.4-18.1	9.7 ± 1.9	19.3	2.4	0.31	0.37	3.4-18.1	9.7 ± 2.2	22.1	2.74	0.47	1.2	0.95^ns^	0.96^ns^	0.01**	0.82^ns^
PB	8.0-194	48.4 ± 22	45.5	27.33	1.33	3.51	8.0-194	49.9 ± 28.5	57.1	33.1	1.67	4.53	0.23^ns^	0.33^ns^	0.00**	0.68^ns^
DFF	31.0-89	49.3 ± 8.8	17.8	11	0.9	0.87	31.0-89	48.1 ± 10.0	20.8	9	0.9	1.11	0.02*	0.03*	0.02*	0.01**
DM	59.0-110	79.9 ± 7.6	9.6	9	0.15	0.1	59.0-105	79.0 ± 9.0	11.4	9.3	0.18	0.35	0.02*	0.04*	0.00**	0.06^ns^
PH	30.4-145.6	71.3 ± 14.1	19.8	19.53	0.23	0.41	30.4-145.6	69.6 ± 17.0	24.5	20.4	0.93	2.84	0.02*	0.05^ns^	0.00**	0.00**
SD	5.2-19.7	11.4 ± 2.2	19.4	2.93	0.4	0.12	5.5-19.7	11.3 ± 2.5	22.1	3.24	0.59	0.68	0.32^ns^	0.37^ns^	0.01**	0.18^ns^
PDL	3.9-27.7	15.0 ± 4.3	28.3	5.7	-0.07	-0.31	4.7-27.7	15.5 ± 4.8	31.3	6.33	0.18	-0.25	0.05^ns^	0.08^ns^	0.00**	0.17^ns^
NPB	1.0-9.0	4.0 ± 1.1	29	1.37	0.27	0.23	1.0-9.0	4.1 ± 1.4	34.5	2	0.53	0.57	0.09^ns^	0.15^ns^	0.00**	0.38^ns^
NPPP	2.0-173.3	45.4 ± 23.8	52.5	30.33	1.02	1.52	2.0-173	48.8 ± 31.6	64.8	40.2	1.22	1.69	0.01**	0.04*	0.00**	0.59^ns^
NPPC	1.0-9.0	4.1 ± 1.3	31.6	1.7	0.44	0.19	1.0-9.0	4.3 ± 1.6	37.9	1.7	0.71	0.25	0.00**	0.00**	0.00**	0.05^ns^
PL	4.6-13.8	7.9 ± 1.2	15.5	1.23	1.27	2.14	4.6-13.8	8.2 ± 1.7	20.8	1.77	1.16	0.92	0.00**	0.00**	0.00**	0.02*
NSPP	6.3-16.3	12.0 ± 1.0	8.5	1.33	-0.53	1.96	6.3-16.3	12.2 ± 1.5	12.1	1.67	-0.35	1.71	0.01*	0.06^ns^	0.00**	0.06^ns^
PWPP	1.0-73.3	18.9 ± 10.3	54.5	13.34	0.8	0.9	1.5-73.3	21.2 ± 13.6	64	17.5	1.03	1.07	0.00**	0.00**	0.00**	0.02*
GY	1.0-46.4	11.7 ± 6.8	57.9	9.13	0.76	0.74	1.0-46.4	13.0 ± 8.7	66.8	11.3	0.98	0.99	0.00**	0.00**	0.00**	0.05*
SW	1.17-8.0	3.3 ± 1.0	29.8	1.05	1.16	1.42	1.4-8.0	3.5 ± 1.3	37.3	1.43	1.12	0.79	0.00**	0.02*	0.00**	0.49^ns^
SA	5.9-28.1	15.9 ± 3.0	19	3.17	1.01	1.19	6.4-28.1	16.3 ± 3.9	23.6	4.47	0.84	0.31	0.01**	0.03*	0.00**	0.37^ns^
SL	3.1-7.2	5.1 ± 0.5	9.9	0.56	0.86	1.12	3.1-7.2	5.2 ± 0.7	12.7	0.74	0.73	0.43	0.02*	0.05*	0.00**	0.48^ns^
SW	2.5-5.4	4.0 ± 0.4	9	0.43	0.5	0.52	2.6-5.4	4.1 ± 0.4	10.7	0.58	0.32	0.16	0.06^ns^	0.11^ns^	0.00**	0.22^ns^
SR	0.6-0.9	0.8 ± 0.03	4.2	0.04	-0.09	0.29	0.7-0.9	0.8 ± 0.03	4.7	0.05	-0.15	0.13	0.33^ns^	0.37^ns^	0.01**	0.48^ns^

SD, Standard Deviation; CV, Coefficient of Variation; IQR, Interquartile Range; traits’ abbreviations are same as in the **Table 1**.

a Newman–Keuls test is used to compare the means of the entire collection and the core set.

b t-test is used to differentiate between the means of the entire collection and the core set.

c Homogeneity of variance between the entire collection and the core set was tested by Levene’s test.

d Differences in frequency distribution tested by the Wilcoxon rank-sum test.

^ns^indicate non-significant; * and ** indicate significant differences at 5% and 1% probability levels, respectively.

**Table 3:** Principal component analysis and explained variability for the respective PCs.

	Entire collection						Core collection (Core E-NE)					
Parameters	PC1	PC2	PC3	PC4	PC5	PC6	PC1	PC2	PC3	PC4	PC5	PC6
Standard deviation	2.30	1.88	1.77	1.48	1.05	1.02	2.33	1.95	1.80	1.48	1.09	1.03
Eigenvalue	5.30	3.53	3.14	2.18	1.10	1.03	5.44	3.80	3.24	2.20	1.18	1.07
Variability (%)	25.24	16.82	14.95	10.37	5.22	4.93	25.92	18.08	15.42	10.49	5.64	5.09
Cumulative variability (%)	25.24	42.05	57.00	67.38	72.59	77.52	25.92	44.00	59.42	69.91	75.54	80.63
Factors loadings												
TLL	-0.14	-0.01	**0.31**	0.44	0.10	-0.08	0.24	0.03	0.16	**0.45**	0.15	-0.03
TLW	-0.16	0.00	0.27	**0.47**	0.10	-0.10	0.24	0.04	0.13	**0.48**	0.17	0.01
PTL	-0.06	-0.05	**0.31**	**0.45**	0.00	-0.07	0.13	0.09	0.17	**0.51**	0.08	0.06
PB	0.05	-0.13	**0.42**	-0.21	-0.02	-0.02	0.01	0.22	**0.41**	-0.14	-0.01	0.07
DFF	**0.28**	0.17	0.21	-0.18	0.01	-0.01	-0.23	-0.16	**0.33**	-0.11	0.01	0.04
DM	**0.26**	0.15	0.21	-0.17	0.01	-0.08	-0.20	-0.13	**0.34**	-0.10	0.07	0.07
PH	0.09	-0.04	**0.31**	0.07	-0.21	0.26	0.01	0.06	**0.30**	0.16	**-0.48**	0.07
SD	0.09	-0.09	**0.42**	-0.18	-0.06	-0.02	0.02	0.18	**0.44**	-0.10	-0.06	0.11
PDL	-0.20	-0.18	-0.15	0.10	-0.16	0.04	0.12	0.20	-0.23	0.03	**-0.33**	0.30
NPB	0.07	-0.19	**0.29**	-0.19	0.03	-0.05	-0.03	0.24	**0.29**	-0.13	0.02	-0.09
NPPP	-0.08	**-0.47**	0.03	-0.14	0.07	-0.13	0.05	**0.46**	-0.02	-0.14	0.14	0.00
NPPC	-0.16	**-0.26**	-0.21	0.11	0.01	-0.09	0.06	0.23	**-0.32**	0.04	0.01	0.26
PL	**-0.33**	0.13	0.05	-0.07	-0.20	0.26	**0.34**	-0.13	0.02	-0.09	-0.29	-0.07
NSPP	-0.13	-0.13	0.06	-0.01	-0.33	**0.73**	0.14	0.14	-0.01	0.02	**-0.62**	**-0.38**
PWPP	-0.23	**-0.39**	0.07	-0.19	0.07	-0.10	0.20	**0.40**	0.01	-0.19	0.16	-0.03
GY	-0.24	**-0.38**	0.05	-0.18	0.09	-0.10	0.21	**0.39**	-0.02	-0.17	0.17	-0.05
SW	**-0.36**	0.18	0.07	-0.14	0.04	-0.01	**0.37**	-0.14	0.03	-0.15	0.01	-0.03
SA	**-0.34**	0.27	0.10	-0.16	0.09	-0.06	**0.36**	-0.22	0.06	-0.16	0.08	-0.01
SL	**-0.34**	0.27	0.10	-0.16	-0.07	-0.15	**0.36**	-0.22	0.08	-0.17	0.07	0.12
SB	**-0.32**	0.24	0.10	-0.16	0.33	0.07	**0.34**	-0.20	0.07	-0.15	0.15	-0.22
SR	0.09	-0.08	-0.01	0.02	0.79	**0.47**	-0.14	0.10	-0.05	0.08	0.11	**-0.77**

Values in bold indicate the traits with the greater contribution to the corresponding principal component.

### 3.2 Frequency distribution of qualitative and quantitative traits

The frequency distributions of 29 descriptor states of seven qualitative characteristics in the 3903 mungbean accessions indicated a wide range of variability (**Figure 4**). The frequency distribution of qualitative traits showed an almost proportionate representation of each descriptor state in the core collection compared to the entire collection (**Figure 4**). The frequency distribution supplements the information given in **Table 1** regarding the structure of mungbean diversity for these qualitative traits. The frequency distributions of the quantitative traits also showed similar patterns in the core collection, as well as the entire collection, indicating good representation of the entire diversity (**Figure 5**). A wide range of variation was observed for most of the quantitative traits, such as PTL, PB, DFF, PH, SD, PDL, NPB, NPPP, NPPC, GY, SW, and SA (**Table 2**; **Figure 5**; **Supplementary Figure S7**).

### 3.3 Sampling of accessions for the core collections development using different methods and comparative evaluation

The multi-environment data indicated a significant difference in terms of error variance for each phenotypic trait studied (**Supplementary information 1** **Supplementary Table S1**). Therefore, the single location phenotypic data for the season 2020 from Jodhpur was selected for further analyses. The basis for selecting this location was its representation of agroclimatic conditions and soil types in the major mungbean growing areas. Moreover, the phenotypic expression of mungbean germplasm at the location over the seasons was relatively better. The phenotypic data on the entire collection (EC) were subjected to core set extraction methods, viz., PowerCore, PCSS, EN100, AN100, and EN50:AN50, with a fixed core size of 400 (approximately 10% of the EC). Each of the core sets was compared with various quality indices to identify the best core set in terms of the quantum of diversity and its representation (**Table 4**). The core sets 1, 3, and 5 showed relatively higher diversity levels and enriched alleles, as highlighted by the E-NE values (i.e., 0.116 and 0.115, respectively). In contrast, core sets 4 and 5 showed a relatively better representation of diversity (A-NE), but lower MD%, VD%, CR%, and VR%. Overall, the performance of the Core set 3 for the quality indices was found superior in terms of diversity and allele richness (**Table 4**). The EN100 method outperformed the other methods, such as AN100 and EN50:AN50 in terms of VR%, CR%, and class coverage. It was also superior to SPSS and PowerCore methods with respect to diversity level (E-NE) and its representation (A-NE) (**Table 4**).

**Table 4:** Comparison of different core sets developed using different statistical evaluation parameters.

Parameters	Core set 1 (PowerCore)	Core set 2 (PCSS)	Core set 3 (EN100)	Core set 4 (AN100)	Core set 5 (EN50:AN50)
E-NE	0.12	0.09	0.14	0.09	0.13
A-NE	0.09	0.10	0.09	0.07	0.08
E-E	0.30	0.29	0.29	0.24	0.27
MD%	71.43	66.67	66.67	9.52	42.86
VD%	100.00	100.00	100.00	42.86	85.71
CR%	99.79	98.01	97.40	90.76	93.29
VR%	123.60	128.60	119.60	108.20	114.20
Class coverage %	100.00	98.57	100.00	98.57	100.00
Sign test (Mean)	0.02*	0.05*	0.02*	0.51^ns^	0.05*
Sign test (Variance)	0.00**	0.00**	0.00**	0.13^ns^	0.00**
Ratio of phenotype retained	1.00	0.97	1.00	0.97	1.00
Mantel correlation	**	**	**	**	**

E-NE, Average distance between each entry and the nearest neighboring entry; A-NE, Average distance between each accession and the nearest entry; E-E, Average genetic distance between entries; MD%, Mean difference percentage; VD%, Variance difference percentage; CR%, Coincidence rate of range; VR%, Variable rate of range; PCSS, Principal component score strategy; EN100, E-NE with 100% weightage; AN100, A-NE with 100% weightage; EN50:AN50, method with equal weightage to E-NE and A-NE.

* and ** indicate significant differences at 5% and 1% probability levels, respectively.

### 3.4 Comparative evaluation of the chosen core collection (EN100) with the entire collection

The comparison of the core collection EN100 with the EC for the range and pattern of diversity across the quantitative and qualitative traits indicated a similar trend (**Table 2**). The phenotypic quantitative trait value ranges are almost the same in the chosen core to the EC, and slight differences are observed in the means of both populations for most of the traits. However, a significant increase in the phenotypic coefficient of variation [CV% (P)] was observed in the core collection (CC) across the traits studied, which was expected to occur due to a decrease in sample size in the core collection (**Table 2**). The IQR range was relatively higher in CC than in EC, except for the trait DFF, indicating the availability of diversity in a wider range of the middle 50% of the distribution. The skewness and kurtosis parameters of the frequency distribution showed slight differences between EC and CC. Comparison of means by the Newman-Keuls test and the t-test indicated that the means of the CC and EC were similar for traits such as TLL, PTL, PB, SD, PDL, NPB, SW, and SR, while for other traits were different (**Table 2**). The Levene’s Test for homogeneity of variance showed significant differences between CC and EC, which was primarily attributed to enhanced variance in CC compared to EC. The Wilcoxon rank-sum test, a non-parametric test, showed that the frequency distributions of 15 quantitative traits, viz., TLL, PTL, PB, DM, SD, PDL, NPB, NPPP, NPPC, NSPP, SW, SA, SL, SW and SR were similar in both the groups, i.e., CC and EC.

**Figure 7:**
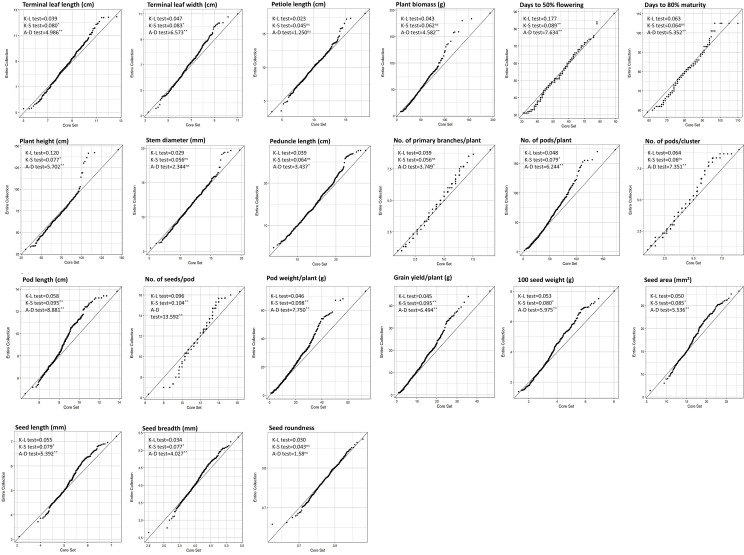
Quantile-Quantile plot along with the Kullback-Leibler distance (K-L), Kolmogorov-Smirnov (K-S), and Anderson-Darling (A-D) goodness of fit test for 21 quantitative data sets of the entire collections and core collection.

The visualization of Q-Q plots showed good representation for the traits viz., PTL, PB, DM, SD, PDL, NPB, NPPC, and SR, while traits such as TLL, TLW, PH, NPPP, PL, NSPP, PWPP, GY, SW, and SA showed deviations in upper quantiles; and the same is also revealed by Kolmogorov-Smirnov (K-S) and Anderson-Darling (A-D) test values shown in the graph (**Figure 7**). Although both the K-S and A-D tests are used for the comparison of the distribution between two samples, the A-D test is considered more powerful if deviations are in the tails of the distribution, as observed in this study (**Figure 7**). The box plot also showed homogeneous distribution of diversity for each trait in CC when compared to the EC. Median value, quartile ranges and outliers are similar in both populations with slight deviations, indicating the representation of diversity as well as conserving the rare allele in CC (**Figure 5**). The bar chart frequency distribution also showed a good representation of the phenotypic diversity of the EC into the CC (**Supplementary Figure S2**).

The CC and EC were also compared for the seven qualitative traits studied using three diversity indices, viz., Shannon’s Diversity Index (H’), Shannon’s Equitability Index (J’), and the maximum potential diversity index (H_max_). A comparison between EC and CC indicated that all the descriptor states available in the EC were represented in the CC for each trait; therefore, H_max_ was the same for both CC and EC (**Table 5**). The values for the other two indices, viz., H’ and J’, are greater for CC than for EC, which is as expected. The frequency distribution of qualitative traits also showed homogeneous distribution in both EC and CC, and representation of all descriptor states for each trait in CC. However, slight deviations in the frequency distribution for a few descriptor states were observed (**Figure 4**).

**Table 5:** Comparison of core collection (CC) with the entire collection (EC) of germplasm using diversity indices for qualitative parameters.

Parameters	No. of descriptor states	Entire Collection (EC)			Core Collection (CC)		
		H’_max_	H’	J’	H’_max_	H’	J’
Hypocotyl Color	5	1.609	1.119	0.695	1.609	1.187	0.737
Seedling Vigor	3	1.099	0.873	0.795	1.099	0.949	0.864
Growth Habit	3	1.099	0.686	0.625	1.099	0.857	0.78
Raceme Position	3	1.099	0.586	0.533	1.099	0.737	0.671
Pod attachment to the peduncle	2	0.693	0.33	0.476	0.693	0.504	0.727
Pod Pubescence	4	1.386	0.689	0.497	1.386	0.844	0.609
Seed Color	9	2.197	1.346	0.612	2.197	1.536	0.667

H’_max_, maximum potential diversity; H, Shannon-Weaver diversity index; J, Evenness (Shannon’ Equitability Index).

### 3.5 Hierarchical clustering, principal component and correlation analysis

The entire mungbean collection, as well as the core collection, was also subjected to dimension reduction-based data analysis to understand the pattern and structure of diversity using agglomerative hierarchical clustering (AHC) and principal component analysis (PCA) methods. AHC segregates accessions into homogenous groups based on similar data points, while PCA basically segregates accessions based on specific patterns and also provides relationships between accessions and traits. The results are useful for identifying desired genotypes of contrasting or similar nature across traits, depending upon the specific objective. Hierarchical clustering further revealed the relationships among the accessions with respect to the quantitative phenotypic data (**Figure 6**). The clustering of accessions showed that the phenotypic variation has no relation to the geographical origin of the mungbean collections (**Figure 6**, **Table 1** of **Supplementary information 1**).

**Figure 8:**
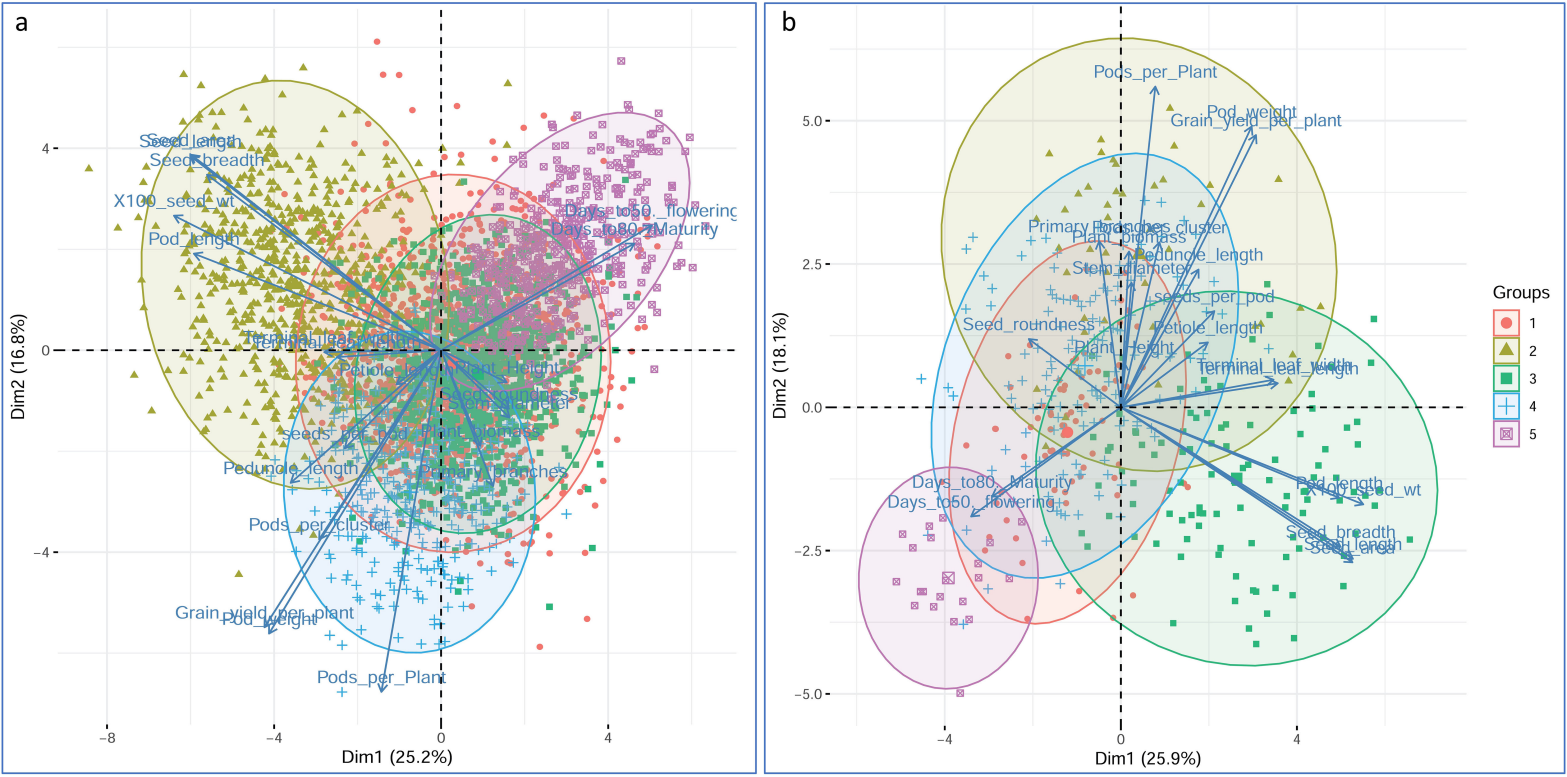
PCA bi-plot for the first two principal components (PCs) derived using quantitative traits data. Total variation explained by the first two PCs is 42% in the entire collection **(a)** and 44% in the core collection **(b)**. For the first two PCs, the grouping of accessions in a two-dimensional plot can be seen, especially for flowering, maturity, and seed dimension-related traits in both **(a, b)**. Accessions and traits falling in the same coordinates indicated that they are correlated to each other, and the variability explained in PCs is mainly attributed to the respective traits. Traits plotted close to each other and distant from the center are positively correlated and vice versa.

PCA was performed using the quantitative data on 21 parameters, and the results revealed the level of variability present in the core and entire collections (**Table 3**; **Supplementary Figures S3**, **S4**). The PCA results indicated a similar pattern of variability among the principal components (PCs) for the entire collection and core collection (**Table 3**; **Supplementary Figures S8**, **S9**). A slightly greater level of variability in the representation of the core collections was primarily due to sample size reduction (**Table 3**). Plotting of genotypes and variables in 2D space for the first two most important PCs highlighted the relationships among the phenotypic traits and accessions (**Figure 8**). The analysis indicated that seed morphometric traits such as seed breadth and seed length, including 100-seed weight and pod length, were strongly associated with each other and negatively correlated with days to flowering and days to maturity under both conditions, i.e., entire and core collections. Terminal leaf length, terminal leaf width, petiole length, plant height, and seed roundness were neutral but might contribute significantly to the differences in other PCs (**Figure 8**).

**Figure 9:**
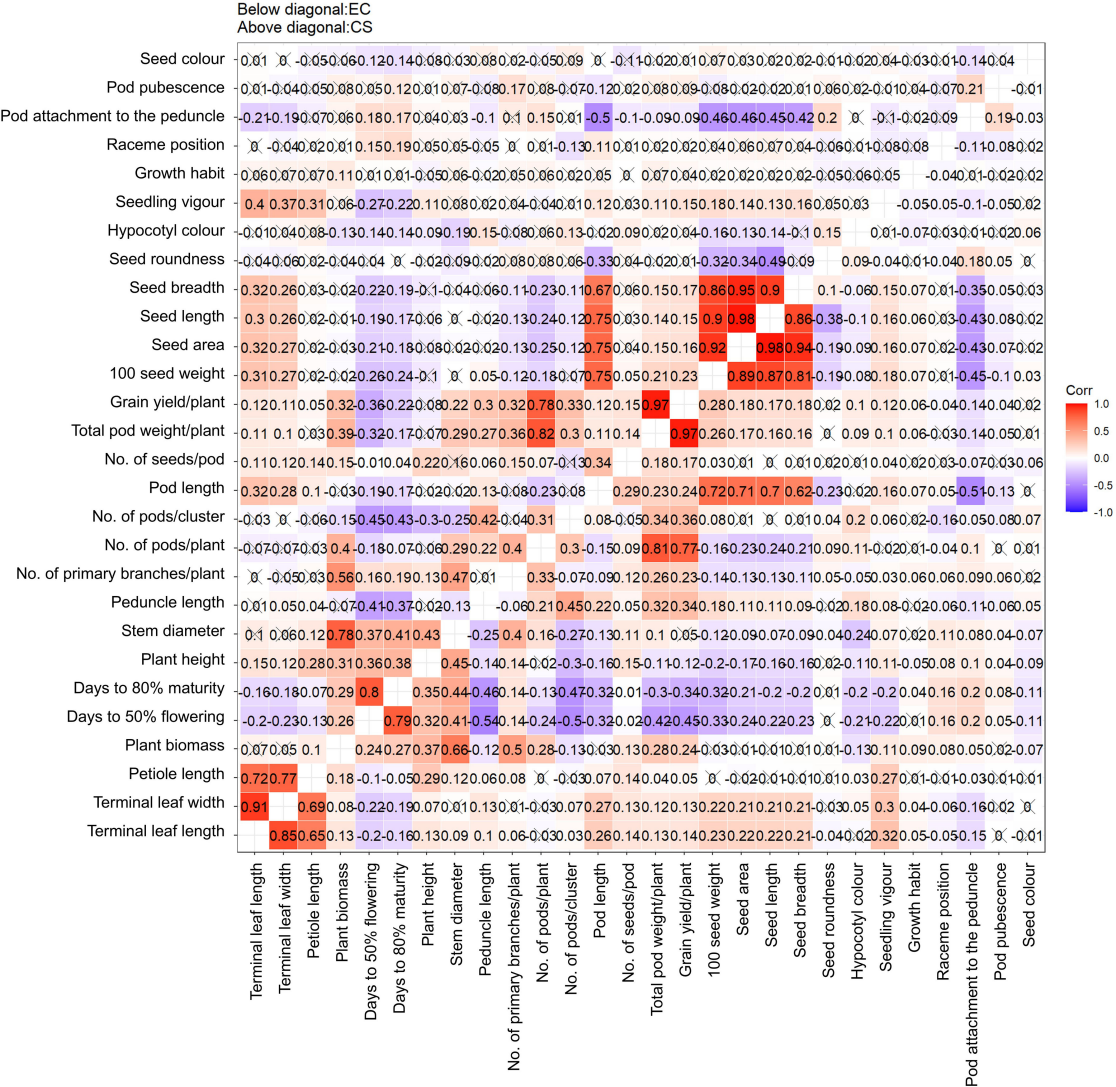
Correlation scatter matrix for quantitative traits for the entire collection and the core collection of mungbean germplasm. The color intensity of each coordinate indicates the magnitude and the direction (+/-) of the correlation. The correlation matrices indicate a similar pattern of correlation among the traits studied for both collections.

The relationships among the phenotypic traits, including quantitative and qualitative, revealed by Pearson’s correlation analysis were similar to those revealed by PCA. The correlation patterns among traits were similar for the EC as well as the CC. A highly significant positive correlation was observed between pod weight/plant and grain yield/plant. Other significant positive correlations were observed between seed morphometric traits (seed area, seed length, and seed width), pod length and 100-seed weight, as expected. The number of pods/plant was positively correlated with the total pod weight/plant and grain yield/plant. The number of days to 50% flowering and the number of days to maturity were negatively correlated with the number of pods/cluster and peduncle length, and other important correlations related to flowering, yield and seedling vigor can be observed in the **Figure 9**.

## Discussion

Plant germplasm collections in genebanks grew exponentially during the early 20^th^ century, but their conservation became increasingly independent of the crop improvement programs since the 1970s. Therefore, to address this, Frankel (1984) and Brown (1989) gave the concept of core collection – “a small subset representing the genetic diversity of the whole collection” for easy management and enhancing germplasm utilization. Early theoretical work based on neutral-allele models predicted that a random 10% core would capture roughly 70% of allelic diversity (Hamon et al., 1994), therefore, this idea is generally used for making a diversity-rich core set of 10% of the entire collection. Further, a large-scale germplasm characterization helps in understanding the level of phenotypic variability and identification of unique and useful traits of breeding importance and the core development activity enhances the utilization of germplasm conserved in genebanks (Odong et al., 2013). Therefore, this study was performed with the same objectives to aid mungbean improvement programs locally as well as globally, as the crop has very high relevance to nutritional and food security (Nair et al., 2013b).

Although the basic purpose of core development remains unchanged, strategies and statistical methods have been continuously changing since the 1980s to maximize diversity richness and its representation in the core collection. Earlier cores developed were primarily based on passport information like the origin of the accession and biological status, phenotypic and biochemical traits data (Bisht et al., 1998; Schafleitner et al., 2015). Later, the sampling method evolved from random sampling to a stratified cluster sampling method following proportional (P), logarithmic (L), constant fraction (C), square root (S), or genetic diversity-dependent allocation (G), to enhance genetic diversity representation and allelic richness (Gu et al., 2023). Schoen and Brown (1993) introduced two other strategies H (Nei’s heterozygosity) and M (maximization), which outperformed earlier P, L, C, and S strategies. Earlier, most of the cores developed followed clustering UPGMA (Unweighted Pair Group Method with Arithmetic Mean) and PCA of the accessions and then sampling within clusters to capture extreme ranges (Gu et al., 2023). Further advances led to the adoption of genetic distance or diversity-based objective functions, such as maximizing allele coverage or minimizing the average distance to the nearest core entry. For example cores can be classified into three categories, viz., a core collection representing all the individual accessions of the entire collection (CC-I), a core collection representing the extremes of the entire collection (CC-X), and a core collection representing the distribution of the entire collection (CC-D) (Odong et al., 2013). The CC-I type represents the entire range of diversity, but the diversity representativeness of the population is lost; the CC-X overrepresents extreme values, therefore lacks representativeness, while CC-D is considered the most optimal method where allele frequencies/distribution are maintained in the core collection. Odong et al. (2013) further proposed three criteria for evaluating a core collection viz., (a) A-NE, average distance between each accession (from the entire collection) and the nearest entry (from the core collection), (b) E-NE, Average distance between each entry and the nearest neighboring entry, and (c) E-E, Average genetic distances between entries.

This study observed that in the studied mungbean germplasm population the E-NE criteria with 100% weightage (EN100) gave the best results. It enhanced the diversity level (E-NE = 0.143), without much compromising on the representation of the diversity (A-NE = 0.091) in comparison to other strategies such as PowerCore, PCSS, AN100, and EN50:AN50 (**Table 4**). The strategy AN100 gave the best representation of the diversity (A-NE = 0.072), but the diversity richness was poor (E-NE = 0.092). Considering these criteria, the core sampling done using the EN100 strategy was selected as the core collection, and further evaluation parameters gave considerable desirable results (**Tables 2**, **3**, **5**; **Figures 4**, **5**, **7**) as discussed below. Maximum representation of the diversity in this core collection will make it a suitable base material for taxonomic studies, trait evaluation, and molecular-based studies for the identification of genes and QTLs.

The results indicated a substantial amount of phenotypic diversity in the mungbean germplasm, as revealed by the summary statistical parameters (**Table 2**) and frequency distributions (**Figures 4**, **5**). This study revealed that traits such as PB, DFF, PH, PDL, NPB, NPPP, GY, and SW can be good target traits for the introgression breeding programs, as highlighted by higher PCV, GCV, and Genetic gains (GG) values (**Table 1**). A higher broad-sense heritability (h²) for a trait indicates that, within the studied population and environments, a larger proportion of the observed phenotypic variation is attributable to genetic differences among genotypes. The results suggest that the traits exhibiting relatively higher H² values such as PB, DFF, DM, PH, SW, PL, PDL, and NSPP, may be useful targets for selection, and could contribute to selection efficiency when combined with appropriate breeding strategies and multi-environment validation (**Table 1**). Similar trait values for these parameters have been reported by other studies in mungbean (Degefa et al., 2014; Xu et al., 2017; Yoseph et al., 2022). The corresponding trait-specific promising accessions identified in this study may be useful in the mungbean breeding program (**Supplementary Table S4**).

The skewness, kurtosis, and frequency distributions (**Table 2**; **Figure 5**) were also similar, with slight deviations, which are primarily due to the sample size reduction and biased selection of accessions to be more useful for breeders. The same is also validated through statistical tests for mean comparison (Newman-Keuls test & t-test), and comparison of frequency distribution (Wilcoxon rank test). Levene’s Test for testing the homogeneity of variance between populations was found significant for all the traits, as a result of sample size reduction (**Table 2**). Nevertheless, the range of variation and deviation indicated that the diversity was well represented in the core set. The diversity indices, viz., Shannon’s Diversity Index (H’), Shannon’s Equitability Index (J’), and maximum potential diversity (H_max_), which were used to evaluate the representation of diversity in the core set for qualitative traits, revealed excellent representation of diversity in the core set (**Table 2**). An increase in H’ and J’ indicated that the level of relative diversity and diversity evenness was greater for the descriptor states for each quality trait. These results indirectly indicate that the core set has a homogenous distribution of variability across the accessions, which augments the usefulness of the core set. For example, genome-wide association studies (GWASs) require a natural, unstructured population with a random distribution of target traits among the individuals of the mapping population (Bush and Moore, 2012). These criteria were fulfilled in the developed core set, making it useful for GWAS. Moreover, as the core set represents the entire collection in a smaller sample size, it becomes a more appropriate resource for the identification of genes/QTLs through the GWAS strategy.

Another mungbean core set was developed on global mungbean collections conserved at AVRDC-The World Vegetable Centre, Taiwan, based on information on the 8 agromorphological characteristics and geographical origin (Schafleitner et al., 2015). However, the mungbean core set developed in this study also included various other parameters, as discussed above. Moreover, the study was developed based on Indian mungbean collections, which are mostly local germplasm collections originating from India. A comparison between two core sets for the mean and variances for common characteristics, viz., plant height, days to 50% flowering, pod length, seeds/pod, and seed weight, indicated substantial differences, which indicated that the mungbean collections conserved in the AVRDC-The World Vegetable Centre and India’s National Genebank were different with respect to their agromorphological characteristics. However, comparison with agro-morphological characterization studies performed on mungbean collection from India’s National Genebank indicated similar results with slight deviations (Bisht et al., 1998; Gayacharan et al., 2020b).

Clustering analyses, viz., hierarchical clustering and principal component analysis (PCA) of the mungbean collections, revealed non-random relationships among the accessions (**Figures 6**, **8**). Clustering helps in the identification and selection of genotypes of contrasting traits with diverse backgrounds and the development of diverse sets. Genotypes from the same cluster may also possess similar traits for abiotic stress tolerance. The hierarchical clustering was performed using a Euclidean distance matrix based on 21 quantitative traits delineated individuals based on the traits (**Figure 6**). It was also observed that the clustering of accessions was not associated with their site of origin (**Supplementary Table S1**), consistent with the results of earlier studies (Yimram et al., 2009; Gayacharan et al., 2020b). These findings indicated that plant origin has the least impact on mungbean structural variability and suggested that mungbean diversity is widely distributed across agroclimatic zones. Most likely, the gene flow in the mungbean population occurs across the mungbean growing region in the country without any geographical hindrance, and the variability must be attributed to local environmental conditions and other selection pressures (Bisht et al., 1998; Gayacharan et al., 2020b).

The variability explained by the first six PCs was comparatively greater for the core set (80.63%) than for the entire collection (77.52%) (**Table 5**). PCA also helped in the identification of useful traits for breeding programs, as revealed by the greater magnitude of loadings in important PCs (Additional information 1). The significant traits identified based on a greater magnitude of vector loading were days to 50% flowering, days to maturity, 100-seed weight, pod length and seed morphometric traits, which are similar to the results explained by h^2^ and GG (**Table 1**). Similar findings were also reported earlier (Gayacharan et al., 2020b); however, the variability explained by the first most important PCs was lower than that in earlier studies, which may be due to the comparatively smaller sample size and biased selection of genotypes in earlier studies (Gayacharan et al., 2020b). In the present study, the first two PCs explained 42% and 44% of the cumulative variability in the EC and CC, respectively. The plotting of traits and accessions in a 2-D plot for the first two PCs revealed a non-random distribution of accessions and relationships between traits (**Figure 8**). The relationships among traits revealed by PCA were similar to the trait relationship patterns revealed by heatmap clustering (**Figure 7**). Furthermore, Pearson’s correlation matrix added information on the magnitude of correlation as well as its direction (**Figure 9**). The correlation among traits helps in the selection of traits for breeding programs. The magnitude of correlation among traits, along with the selection of corresponding useful genotypes from PCA and AHC analysis, helped in the efficient selection of parent genotypes, which resulted in a successful breeding program. The correlations among traits were as expected and were similar under both conditions, viz., EC and CC, which further reiterated the excellent representation of variability in the core set of the entire genebank collections.

## Conclusions

The core collections are among the most important resources for crop improvement programs. The 400 mungbean accessions, which were derived as a core set of the entire mungbean collections conserved in India’s National Genebank, were found to have excellent representation of the genetic variability of the entire collection, as highlighted by the statistical parameters discussed above. The intrinsic values of this core set are heightened by the fact that mungbean is indigenous to India, and the collections used in this study originated from each geographic region of the country; therefore, they represent mungbean diversity well. The findings of this study will have several implications for crop improvement, such as genotype and trait selection, based on the relatively high breeding values of crops, the use of a natural unstructured population for GWAS, and the identification of primary targets for identifying sources of plants against biotic and abiotic stresses and nutritional traits. These findings will also be useful in the development of niche-specific mungbean ideotypes for better morphological characteristics and phenological and yield traits. Therefore, we anticipate that the utilization of the mungbean core set and the other information generated will have tremendous impacts on mungbean breeding programs.

## Data Availability

The original contributions presented in the study are included in the article/**Supplementary Material**. Further inquiries can be directed to the corresponding author.
