# Supplementary material for: Unravelling phenotypic variations and establishing a core collection in mungbean for accelerating the crop improvement programs
Source: Front Plant Sci. 2026 Feb 25;17:1743562. doi: 10.3389/fpls.2026.1743562 (PMC12975986; doi:10.3389/fpls.2026.1743562)
Supplement: Supplementary file 2 [file DataSheet1.docx]

**Table S1:** Basic Passport information for mungbean core collection (CC)

| **Sr. No.** | **Cluster no. (Fig. 7)** | **Accession ID** | **Date of Collection** | **Collector/Other identity** | **State Name** | **Country name** |
| --- | --- | --- | --- | --- | --- | --- |
| 1 | Ia | EC396394 | 1997 |  |  | Unknown |
| 2 | Ia | EC396401 | 1997 |  |  | Unknown |
| 3 | Ia | IC24795 | 06/12/1974 |  | Andhra Pradesh | India |
| 4 | Ia | IC606545 | 2002 |  | Unknown | India |
| 5 | Ia | EC528093 | 2009 | MG 61-2 |  | Japan |
| 6 | Ia | EC528607 | 2009 | VC 6370 (30-65) |  | China |
| 7 | Ia | EC590222 | 2009 | ACC 11008 |  | Sri Lanka |
| 8 | Ia | EC592167 | 2009 | KPS1 |  | Taiwan |
| 9 | Ia | IC396798 | 27/04/2003 | NKSG-88 | Madhya Pradesh | India |
| 10 | Ia | EC396409 | 1997 |  |  | Unknown |
| 11 | Ia | EC396411 | 1997 |  |  | Unknown |
| 12 | Ia | EC396122 | Nov-97 |  |  | Unknown |
| 13 | Ia | EC396410 | 1997 |  |  | Unknown |
| 14 | Ia | EC398901 | Nov-97 | VC 6153B-19 |  | Thailand |
| 15 | Ia | EC396125 | Nov-97 |  |  | Unknown |
| 16 | Ia | EC396156 | Nov-97 |  |  | Unknown |
| 17 | Ia | IC607146 | Nov-97 |  | Unknown | India |
| 18 | Ia | EC251967-1 | 1993 |  |  | Taiwan |
| 19 | Ia | EC272458 | 1993 | VC-3890A |  | Taiwan |
| 20 | Ia | EC314301 | 1993 | VC-4152 A |  | Taiwan |
| 21 | Ia | EC396402 | 1997 |  |  | Unknown |
| 22 | Ia | IC118994 | 1993 | BH-1, PI 363393 | Maharashtra | India |
| 23 | Ia | IC325787 | 12/10/2001 | KCM/BR-71 | Rajasthan | India |
| 24 | Ia | IC332332 | 11/11/2001 | U-53/VR/163 | Madhya Pradesh | India |
| 25 | Ia | IC338858 | 22/11/2001 | IN-53 | Madhya Pradesh | India |
| 26 | Ia | IC338922 | 25/11/2001 | IN-117 | Madhya Pradesh | India |
| 27 | Ia | EC592176 | 2009 | VC 6173B-6 |  | Taiwan |
| 28 | Ia | IC418452 | 21/11/2003 | MTS-37 | Andaman & Nicobar Islands | India |
| 29 | Ia | IC530554 | 19/10/2005 | HUM-16 | Uttar Pradesh | India |
| 30 | Ia | IC571727 | 19/05/2009 | BM 2003-2 | Maharashtra | India |
| 31 | Ia | IC607183 | 2009 |  | Unknown | India |
| 32 | Ia | IC607185 | 2009 |  | Unknown | India |
| 33 | Ia | IC610282 | 03/12/2013 | KVSA-1775 | Andhra Pradesh | India |
| 34 | Ia | IC611649 | 03/03/2012 | ANM-12-01 | Andaman and Nicobar Islands | India |
| 35 | Ia | IC626176 | 2018 | LRM/13-04 | Tamil Nadu | India |
| 36 | Ia | IC626181 | 2018 | LRM/13-10 | Tamil Nadu | India |
| 37 | Ia | EC396133 | Nov-97 |  |  | Unknown |
| 38 | Ia | EC396141 | Nov-97 |  |  | Unknown |
| 39 | Ia | EC396530 | 1997 |  |  | Unknown |
| 40 | Ia | EC396113 | Nov-97 |  |  | Unknown |
| 41 | Ia | EC396116 | Nov-97 |  |  | Unknown |
| 42 | Ia | EC398882 | Nov-97 | VC 6153B-20P |  | Thailand |
| 43 | Ia | EC398898 | Nov-97 | VC 6144-B-10 |  | Thailand |
| 44 | Ia | EC398917 | Nov-97 | 6148(50-12) |  | Thailand |
| 45 | Ia | EC396104 | Nov-97 |  |  | Unknown |
| 46 | Ia | IC76569 | 31/12/1967 | M-919 | Delhi | India |
| 47 | Ia | EC396143 | Nov-97 |  |  | Unknown |
| 48 | Ia | EC511376 | 2005 | TV 1590, VC 3920A |  | Taiwan |
| 49 | Ia | IC119032 | 1993 | NKG-138A | Maharashtra | India |
| 50 | Ia | IC39591 | 12/09/1980 | 651/5 | Gujarat | India |
| 51 | Ia | EC398884 | Nov-97 | VC 6173B-10 |  | Thailand |
| 52 | Ib | IC682 | 10/04/1951 | No.T-1 | Unknown | India |
| 53 | Ib | IC30938 | 18/12/1977 | 114 | Tripura | India |
| 54 | Ib | IC113965 | 18/09/1991 | K-4275 | Rajasthan | India |
| 55 | Ib | IC325929 | 31/10/2001 | PNB-5 | Maharashtra | India |
| 56 | Ib | IC332214 | 04/11/2001 | U-53/VR/44 | Madhya Pradesh | India |
| 57 | Ib | IC336750 | 06/11/2001 | VR-22 | Madhya Pradesh | India |
| 58 | Ib | IC338852 | 21/11/2001 | IN-47 | Madhya Pradesh | India |
| 59 | Ib | IC282108 | 22/10/2000 | CN-8096 | Andhra Pradesh | India |
| 60 | Ib | IC613779 | 07/05/2015 | WGG-42 | Telangana | India |
| 61 | Ib | IC488795 | 1994 | PLM-297 | Unknown | India |
| 62 | Ib | IC507476 |  | PLM-718, PI 363920 | Gujarat | India |
| 63 | Ib | IC148540 | 1995 | NIC-7915 | Maharashtra | India |
| 64 | Ib | IC282121 | 23/10/2000 | CN-9028 | Andhra Pradesh | India |
| 65 | Ib | EC396397 | 1997 |  |  | Unknown |
| 66 | Ib | IC76497 | 31/12/1967 | M-700 | Delhi | India |
| 67 | Ib | EC396150 | Nov-97 |  |  | Unknown |
| 68 | Ib | EC520039 |  | VC 6372(45-8-1) |  | China |
| 69 | Ib | EC16563-2 | 1992 |  |  | Sri Lanka |
| 70 | Ib | EC93219 | 1988 |  |  | USA |
| 71 | Ib | EC251786 | 1993 | VC - 3004 A |  | Taiwan |
| 72 | Ib | EC313926 | 1993 | Pagasa-7 |  | Thailand |
| 73 | Ib | EC396404 | 1997 |  |  | Unknown |
| 74 | Ib | IC103821 | 14/10/1989 | DCB-1287 | Gujarat | India |
| 75 | Ib | IC103878 | 30/06/1989 | DCB-1344 | Gujarat | India |
| 76 | Ib | IC118956 | 1993 | CH-6/1 | Maharashtra | India |
| 77 | Ib | IC121193 | 1992 | PLM-186 | Unknown | India |
| 78 | Ib | IC148516 | 13/09/1992 | STV-2831, NIC-15239 | Andhra Pradesh | India |
| 79 | Ib | IC282083 | 19/10/2000 | CN-8052 | Andhra Pradesh | India |
| 80 | Ib | IC282085 | 19/10/2000 | CN-8054 | Andhra Pradesh | India |
| 81 | Ib | IC331228 | 31/10/2001 | IP01-400 | Madhya Pradesh | India |
| 82 | Ib | IC338882 | 23/11/2001 | IN-77 | Madhya Pradesh | India |
| 83 | Ib | EC528091 | 2009 | GWALIER 18 |  | Japan |
| 84 | Ib | IC610 |  |  | Unknown | India |
| 85 | Ib | IC436534 | 10/09/2003 | KARS-030 | Andhra Pradesh | India |
| 86 | Ib | IC436548 | 10/09/2003 | KARS-044 | Andhra Pradesh | India |
| 87 | Ib | IC616500 | 06/10/2010 | NSJ/NAIP/93 | Telangana | India |
| 88 | Ib | IC507414 |  | PLM-477 | Punjab | India |
| 89 | Ib | IC507471 | 1987 | PLM-707 | Gujarat | India |
| 90 | Ib | IC258602 | 16/09/1999 | LB/19 | Maharashtra | India |
| 91 | Ib | IC273267 | 07/10/1999 | AKS-47 | Madhya Pradesh | India |
| 92 | Ib | IC76482 | 31/12/1967 | M-669 | Delhi | India |
| 93 | Ib | EC538119 |  | VC 6369(53-97) |  | Taiwan |
| 94 | Ib | EC538123 |  | KPS 2 |  | Taiwan |
| 95 | Ib | EC9129 |  |  |  | Unknown |
| 96 | Ib | IC8917 | 19/12/1961 | Maru-15 | Rajasthan | India |
| 97 | Ib | IC314527 | 01/03/2000 | PLM-0285 | Madhya Pradesh | India |
| 98 | Ib | IC338902 | 24/11/2001 | IN-97 | Madhya Pradesh | India |
| 99 | Ib | IC488879_1 | 1988 | PLM-324 | Bihar | India |
| 100 | Ib | IC507291 | 1988 | PLM-116, PI 363421 | Bihar | India |
| 101 | Ib | IC554399 | 30/08/2007 | EC-16569-A-1 | Unknown | India |
| 102 | Ib | IC329078 | 13/10/2001 | OPY141 | Rajasthan | India |
| 103 | Ib | IC488942 |  | PLM-473 | Punjab | India |
| 104 | Ib | IC436633 | 11/10/2003 | KARS-134 | Andhra Pradesh | India |
| 105 | Ib | IC436726 | 15/10/2003 | KARS-243 | Andhra Pradesh | India |
| 106 | Ib | IC426772 | 12/03/2003 | BAR-031 | Andhra Pradesh | India |
| 107 | Ib | IC5482-2 |  |  | Unknown | India |
| 108 | IIa | IC400162 | 14/11/2002 | KCM-612 | Odisha | India |
| 109 | IIa | EC245949 | 1993 | VC 3902 A |  | Taiwan |
| 110 | IIa | IC52078 | 08/03/1982 | TR 276/5 | Haryana | India |
| 111 | IIa | IC102913 | 27/10/1986 | DCB-185 | Rajasthan | India |
| 112 | IIa | IC119035 | 2002 | U13/199 | Maharashtra | India |
| 113 | IIa | IC121262 | 1992 | PLM-703 | Unknown | India |
| 114 | IIa | IC541818 | 01/10/2005 | PLM-471 | Unknown | India |
| 115 | IIa | IC507281 |  | PLM-97, PI 363402 | Madhya Pradesh | India |
| 116 | IIa | IC507459 | 1987 | PLM-645 | Gujarat | India |
| 117 | IIa | IC507530 | 1988 | PLM-997 | Gujarat | India |
| 118 | IIa | IC11365 | 25/11/1964 |  | Gujarat | India |
| 119 | IIa | EC512781 |  | PI-473916 |  | USA |
| 120 | IIa | IC39500 | 12/09/1980 | 464/4 | Gujarat | India |
| 121 | IIa | IC43600 | 10/07/1981 | No. 303 | Karnataka | India |
| 122 | IIa | IC121177 | 1992 | PLM-44, PI 363351 | Unknown | India |
| 123 | IIa | IC325788 | 12/10/2001 | KCM/BR-72 | Rajasthan | India |
| 124 | IIa | IC600516 | 1988 |  | Unknown | India |
| 125 | IIa | IC73532 | 01/03/2000 | ML-267 | Punjab | India |
| 126 | IIa | IC415625 | 14/10/2003 | IP-14 | Maharashtra | India |
| 127 | IIa | IC424988 | 25/05/2004 | PLM-138, PI-363442 | Delhi | India |
| 128 | IIa | IC424989 | 25/05/2004 | PLM-173, PI-363474 | Delhi | India |
| 129 | IIa | IC436763 | 15/10/2003 | KARS-279 | Andhra Pradesh | India |
| 130 | IIa | IC589144 | 26/07/2011 | VGGru 1 | Tamil Nadu | India |
| 131 | IIa | IC507342 | 1988 | PLM-234 | Bihar | India |
| 132 | IIa | IC259531 | 09/10/1999 | IP99-30 | Goa | India |
| 133 | IIa | IC11296 | 25/11/1964 |  | Gujarat | India |
| 134 | IIa | EC27515 | 1986 | 305 FC no. 4412 |  | Pakistan |
| 135 | IIa | IC8941-2 | 19-12-1961 |  | Rajasthan | India |
| 136 | IIa | IC39382 | 12/08/1980 | 248/7 | Rajasthan | India |
| 137 | IIa | IC39391 | 12/08/1980 | 323/2 | Rajasthan | India |
| 138 | IIa | IC39438 | 12/09/1980 | 396/2 | Rajasthan | India |
| 139 | IIa | IC39444 | 12/09/1980 | 403/1 | Rajasthan | India |
| 140 | IIa | IC39318 | 12/08/1980 | 107/2 | Rajasthan | India |
| 141 | IIa | IC39549 | 12/09/1980 | 510/5 | Gujarat | India |
| 142 | IIa | IC39564 | 12/09/1980 | 553/1 | Gujarat | India |
| 143 | IIa | IC39576 | 12/09/1980 | 618/2 | Gujarat | India |
| 144 | IIa | IC80156 | 01/04/1982 | CH-10/1 | Rajasthan | India |
| 145 | IIa | IC314595 | 01/03/2000 | PLM-0379 | Jammu and Kashmir | India |
| 146 | IIa | IC472099 | 28/04/2005 | Bhakrani | Kerala | India |
| 147 | IIa | IC488601 | 1993 | BANDA/P1 | Unknown | India |
| 148 | IIa | IC488604 | 1991 | CH-1-1 | Unknown | India |
| 149 | IIa | IC488855 | 1988 | PLM-660, | Gujarat | India |
| 150 | IIa | IC488920_1 | 1988 | PLM-942 | Gujarat | India |
| 151 | IIa | IC488962 | 1988 | PLM-953 | Gujarat | India |
| 152 | IIa | IC489061 | 1988 | PLM-989 | Gujarat | India |
| 153 | IIa | IC311437 | 29/10/2002 | ASA-46 | Gujarat | India |
| 154 | IIa | IC314925 | 01/03/2000 | PLM-0444 | Punjab | India |
| 155 | IIa | IC472116 | 28/04/2005 | Nswera | Kerala | India |
| 156 | IIa | IC472118 | 28/04/2005 | Pal | Kerala | India |
| 157 | IIa | IC541834 | 01/10/1986 | N-440 | Unknown | India |
| 158 | IIa | IC541837 | 01/10/1986 | Salawas/P15 | Rajasthan | India |
| 159 | IIa | IC488833 | 1994 | PLM-141 | Unknown | India |
| 160 | IIa | IC488738 | 1994 | PLM-366 | Unknown | India |
| 161 | IIa | IC121290 | 1992 | PLM-897 | Unknown | India |
| 162 | IIa | IC121301 | 1992 | PLM-897 | Unknown | India |
| 163 | IIa | IC507533 | 1988 | PLM-1014 | Gujarat | India |
| 164 | IIa | IC118998 | 1993 | COGB-2 | Maharashtra | India |
| 165 | IIa | EC249656 | 1993 | OR VO4330B - BR |  | Taiwan |
| 166 | IIa | IC541817 | 01/10/2005 | PLM-97A | Unknown | India |
| 167 | IIa | IC488554_1 |  | PLM-578, PI 363802 | Punjab | India |
| 168 | IIa | IC489114_1 | 1993 | PLM-424 | Haryana | India |
| 169 | IIa | IC399198 | 04/03/2003 | TNAU10008 | Tamil Nadu | India |
| 170 | IIa | IC76389 | 1987 | M-487 | Unknown | India |
| 171 | IIa | IC76479 | 31/12/1967 | M-665 | Delhi | India |
| 172 | IIa | IC11445-5 | 1988 |  | Gujarat | India |
| 173 | IIa | IC39487 | 12/09/1980 | 454/3 | Gujarat | India |
| 174 | IIa | IC39459 | 12/09/1980 | 423/1 | Gujarat | India |
| 175 | IIa | IC39560 | 12/09/1980 | 530/4 | Gujarat | India |
| 176 | IIa | IC113985 | 18/09/1991 | DCB-170 | Rajasthan | India |
| 177 | IIa | IC370739 | 27/09/2002 | OPY-169 | Rajasthan | India |
| 178 | IIa | IC426771 | 12/03/2003 | BAR-022 | Andhra Pradesh | India |
| 179 | IIa | IC489101 |  | PLM-502, PI 363742 | Punjab | India |
| 180 | IIa | IC488725 | 1991 | DCB-610 | Unknown | India |
| 181 | IIa | IC488812 | 1991 | SALWAS/P11 | Unknown | India |
| 182 | IIa | IC565295 | 11/04/2008 | DP-21/108 | Odisha | India |
| 183 | IIa | IC257571 | 14/12/1999 | RM4/U48/DP-93 | Odisha | India |
| 184 | IIa | IC331182 | 05/11/2001 | KG/ZH/BLM-266 | Gujarat | India |
| 185 | IIa | IC343864 | 23/12/2001 | SKN-055 | Andhra Pradesh | India |
| 186 | IIa | IC488657 | 1991 | CH-119-2 | Unknown | India |
| 187 | IIa | EC309456_1 | 1993 | PLM-618, V-4483 |  | Taiwan |
| 188 | IIa | IC343878 | 23/12/2001 | SKN-069 | Andhra Pradesh | India |
| 189 | IIa | IC436764 | 15/10/2003 | KARS-280 | Andhra Pradesh | India |
| 190 | IIa | IC436810 | 16/10/2003 | KARS-332 | Andhra Pradesh | India |
| 191 | IIa | IC436813 | 16/10/2003 | KARS-335 | Andhra Pradesh | India |
| 192 | IIa | IC488794 | 1994 | PLM-513 | Unknown | India |
| 193 | IIa | IC76366 | 1987 | M-455 | Unknown | India |
| 194 | IIa | EC16556 |  |  |  | Unknown |
| 195 | IIa | IC39476 | 12/09/1980 | 445/1 | Gujarat | India |
| 196 | IIa | IC39485 | 12/09/1980 | 452/4 | Gujarat | India |
| 197 | IIa | IC39511 | 12/09/1980 | 474/3 | Gujarat | India |
| 198 | IIa | IC39523 | 12/09/1980 | 485/5 | Gujarat | India |
| 199 | IIa | IC39464 | 12/09/1980 | 429/10 | Gujarat | India |
| 200 | IIa | IC39577 | 12/09/1980 | 620/4 | Gujarat | India |
| 201 | IIa | IC488809 | 1991 | CH-119-5 | Unknown | India |
| 202 | IIa | IC52070 | 08/03/1982 | TR 158/1 | Rajasthan | India |
| 203 | IIb | EC249652-2 | 1993 | OR VO4229A - G |  | Taiwan |
| 204 | IIb | EC272454 | 1993 | VC-3301A |  | Taiwan |
| 205 | IIb | EC314302 | 1993 | VC-4117 A |  | Taiwan |
| 206 | IIb | IC8592 | 23/10/1961 |  | Himachal Pradesh | India |
| 207 | IIb | IC8854 | 19/12/1961 |  | Rajasthan | India |
| 208 | IIb | IC10483-3 |  |  | Unknown | India |
| 209 | IIb | IC11438-4 | 25.11.1964 |  | Gujarat | India |
| 210 | IIb | IC121298 | 1992 | PLM-957 | Unknown | India |
| 211 | IIb | IC121315 | 1992 | PS-8 | Unknown | India |
| 212 | IIb | IC148444 | 09/09/1992 | STV-2720, NIC-15163 | Andhra Pradesh | India |
| 213 | IIb | EC581523 | 2009 | PGRC 10034, Ari mung |  | Sri Lanka |
| 214 | IIb | IC73359 | 31/12/1985 | DMS-1 | Bihar | India |
| 215 | IIb | IC395518 | 30/07/2003 | TARM-18 | Unknown | India |
| 216 | IIb | IC436543 | 10/09/2003 | KARS-039 | Andhra Pradesh | India |
| 217 | IIb | IC470530 | 06/11/2004 | SM/BC-120 | Rajasthan | India |
| 218 | IIb | IC488897 |  | PLM-414, PI 363671 | Punjab | India |
| 219 | IIb | IC489046 | 1988 | PLM-823 | Rajasthan | India |
| 220 | IIb | EC397140 | 2002 |  |  | Unknown |
| 221 | IIb | EC251768 | 1993 | VC - 2768 A |  | Taiwan |
| 222 | IIb | IC11332 | 25/11/1964 |  | Gujarat | India |
| 223 | IIb | IC52079 | 08/03/1982 | TR 308/7 | Rajasthan | India |
| 224 | IIb | IC103975 | 21/10/1989 | DCB-1441 | Gujarat | India |
| 225 | IIb | IC338883 | 23/11/2001 | IN-78 | Madhya Pradesh | India |
| 226 | IIb | IC648-5 |  |  | Unknown | India |
| 227 | IIb | IC148531 | 1995 | SV-2438 | Maharashtra | India |
| 228 | IIb | IC600183 | Nov-87 |  | Unknown | India |
| 229 | IIb | IC548369 |  |  | Unknown | India |
| 230 | IIb | IC754464 |  |  | Unknown | India |
| 231 | IIb | EC260603 | 1993 | VC 1974 A/VC 2770A |  | Taiwan |
| 232 | IIb | IC76338 | 1987 | M-401 | Unknown | India |
| 233 | IIb | IC488775 |  | PLM-169, PI 363470 | Unknown | India |
| 234 | IIb | IC39448 | 12/09/1980 | 407/5 | Gujarat | India |
| 235 | IIb | IC39493 | 12/09/1980 | 457/2 | Gujarat | India |
| 236 | IIb | IC52074 | 08/03/1982 | TR 240/4 | Haryana | India |
| 237 | IIb | IC121200 | 1992 | PLM-252 | Unknown | India |
| 238 | IIb | IC148528 | 1995 | MG-34 | Maharashtra | India |
| 239 | IIb | IC323998 | 02/10/2001 | PDA-17 | Gujarat | India |
| 240 | IIb | IC311409 | 25/10/2002 | ASA-18 | Gujarat | India |
| 241 | IIb | IC507276 | 1988 | PLM-38 | Bihar | India |
| 242 | IIb | IC520034 |  |  | Unknown | India |
| 243 | IIb | IC39399 | 12/08/1980 | 334/8 | Gujarat | India |
| 244 | IIb | IC39415 | 12/08/1980 | 370/4 | Gujarat | India |
| 245 | IIb | IC52058 | 08/03/1982 | TR 70/1 | Rajasthan | India |
| 246 | IIb | IC341273 | 26/11/2000 | LBa-74 | Maharashtra | India |
| 247 | IIb | IC489076_1 | 1988 | PLM-743, PI 363945 | Gujarat | India |
| 248 | IIb | IC2029 | 01/03/1953 | T54 | Unknown | India |
| 249 | IIb | IC10502 | 10/01/1963 | PI 363268 | Gujarat | India |
| 250 | IIb | IC10843 | 05/03/1964 | G.G.525 | Andhra Pradesh | India |
| 251 | IIb | IC24782 | 06/12/1974 |  | Andhra Pradesh | India |
| 252 | IIb | IC39547 | 12/09/1980 | 508/5 | Gujarat | India |
| 253 | IIb | IC113983 | 18/09/1991 | NKG-138-A | Rajasthan | India |
| 254 | IIb | IC119001 | 1993 | CH-6/1 | Maharashtra | India |
| 255 | IIb | IC119026 | 2002 | Mura-122 | Maharashtra | India |
| 256 | IIb | IC119106 | 2002 | Lihulst-P1 | Maharashtra | India |
| 257 | IIb | IC73533 | 01/03/2000 | ML-131 | Punjab | India |
| 258 | IIb | IC73536 | 01/03/2000 | SML-32 | Punjab | India |
| 259 | IIb | IC121318 | 1992 | THAJWAL-BHUNKI/P-1 | Unknown | India |
| 260 | IIb | IC73537 | 01/03/2000 | G-65 | Punjab | India |
| 261 | IIb | IC148384 | 08/01/1995 | KM-5502, NIC-22824 | Goa | India |
| 262 | IIb | IC148530 | 1995 | N-1535 | Maharashtra | India |
| 263 | IIb | IC314609 | 01/03/2000 | PLM-0666 | Rajasthan | India |
| 264 | IIb | IC330875 | 21/10/2001 | IP01-353 | Uttar Pradesh | India |
| 265 | IIb | IC330881 | 21/10/2001 | IP01-359 | Uttar Pradesh | India |
| 266 | IIb | IC148541 | 1995 | NIC-7916(LOCAL) | Maharashtra | India |
| 267 | IIb | IC355610 | 25/08/2002 | AKP/NP/8/9 | Bihar | India |
| 268 | IIb | IC398984 | 19/02/2003 | SK-37 | Andhra Pradesh | India |
| 269 | IIb | IC447795 | 29/11/2004 | VKG-30/51 | Jharkhand | India |
| 270 | IIb | IC447908 | 30/10/2004 | VKG-29/28 | Bihar | India |
| 271 | IIb | IC472065 | 28/04/2005 | U-13-125 | Kerala | India |
| 272 | IIb | IC488941 | 1988 | PLM-851, PI 364030 | Rajasthan | India |
| 273 | IIb | IC507242 | 1988 | PLM-12, PI 363318 | Uttar Pradesh | India |
| 274 | IIb | IC507272 | 1988 | PLM-71 | Bihar | India |
| 275 | IIb | IC605755 | 2006 |  | Unknown | India |
| 276 | IIb | IC683-3 |  |  | Unknown | India |
| 277 | IIb | IC8422 |  |  | Unknown | India |
| 278 | IIb | IC385717 | 09/02/2003 | VKS-15/8 | Bihar | India |
| 279 | IIb | IC8971-1 | 19.12.1961 |  | Rajasthan | India |
| 280 | IIb | IC9127-1 | 1987 |  | Rajasthan | India |
| 281 | IIb | IC436668 | 12/10/2003 | KARS-173 | Andhra Pradesh | India |
| 282 | IIb | IC617779 | 2016 | PTP/DC/AMP-25 | Gujarat | India |
| 283 | IIb | IC507272_1 | IC507272_1 | PLM-16 | Bihar | India |
| 284 | IIb | IC488778 | 1994 | PLM-89 | Unknown | India |
| 285 | IIb | IC507242_1 | 1988 | PLM-12, PI 363318 | Uttar Pradesh | India |
| 286 | IIb | IC507399 | 1988 | PLM-394 | Punjab | India |
| 287 | IIb | PLM-607 |  |  | Unknown | India |
| 288 | IIb | PLM-666 |  |  | Unknown | India |
| 289 | IIb | IC489062 |  | PLM-775, PI 363972 | Rajasthan | India |
| 290 | IIb | IC488568 | 1988 | PLM-1024 | Unknown | India |
| 291 | IIb | IC472062 | 28/04/2005 | S-9 | Kerala | India |
| 292 | IIb | IC282133 | 24/10/2000 | CN-9061 | Andhra Pradesh | India |
| 293 | IIb | IC282155 | 22/10/2000 | CN-8089 | Andhra Pradesh | India |
| 294 | IIb | IC283542 | 01/12/2000 | VKS/SCC-4/87 | Bihar | India |
| 295 | IIb | IC297633 | 21/10/2001 | IP2K296 | Punjab | India |
| 296 | IIb | IC313547 | 10/02/2001 | NDS-47 | Bihar | India |
| 297 | IIb | IC507498_1 | 1988 | PLM-794 | Himachal Pradesh | India |
| 298 | IIb | EC396142 | Nov-97 |  |  | Unknown |
| 299 | IIb | IC76398 | 1987 | M-508 | Unknown | India |
| 300 | IIb | IC53778 | 08/02/1982 | P/660 | Madhya Pradesh | India |
| 301 | IIb | IC76460 | 31/12/1967 | M-633 | Delhi | India |
| 302 | IIb | IC76380 | 1987 | M-477 | Unknown | India |
| 303 | IIb | IC76492 | 31/12/1967 | M-688 | Delhi | India |
| 304 | IIb | IC102954 | 30/06/1986 | DCB-241 | Rajasthan | India |
| 305 | IIb | EC13079-2 | 1988 | K-3476 |  | Unknown |
| 306 | IIb | EC246508 | 1993 | V-2802A |  | Taiwan |
| 307 | IIb | EC249640 | 1993 | OR BO2402A - G |  | Taiwan |
| 308 | IIb | EC251555 | 1993 | V - 3476 |  | Taiwan |
| 309 | IIb | EC251557 | 1993 | V - 6017 |  | Taiwan |
| 310 | IIb | IC10932-5 | Nov-87 | G.G.-525 | Andhra Pradesh | India |
| 311 | IIb | IC13080-3 |  |  | Unknwon | India |
| 312 | IIb | IC22478 | 02/01/1974 |  | Madhya Pradesh | India |
| 313 | IIb | IC39293 | 12/08/1980 | 47/1 | Rajasthan | India |
| 314 | IIb | IC39454 | 12/09/1980 | 415/1 | Gujarat | India |
| 315 | IIb | IC113986 | 18/09/1991 | Pal Village/P3 | Rajasthan | India |
| 316 | IIb | IC118954 | 1993 | K-3991 | Maharashtra | India |
| 317 | IIb | IC118979 | 1993 | U20-38 | Maharashtra | India |
| 318 | IIb | IC121175 | 1992 | PLM-23-A | Unknown | India |
| 319 | IIb | IC121190 | 1992 | PLM-180 | Unknown | India |
| 320 | IIb | IC121220 | 1992 | PLM-459 | Unknown | India |
| 321 | IIb | IC148383 | 07/01/1995 | KM-5499, NIC-22823 | Goa | India |
| 322 | IIb | IC148397 | 06/09/1992 | STV-2624, NIC-15104 | Andhra Pradesh | India |
| 323 | IIb | IC148457 | 10/09/1992 | STV-2738, NIC-15176 | Telangana | India |
| 324 | IIb | IC148474 | 10/09/1992 | STV-2760, NIC-15194 | Andhra Pradesh | India |
| 325 | IIb | IC148494 | 11/09/1992 | STV-2786, NIC-15214 | Andhra Pradesh | India |
| 326 | IIb | IC280489 | 16/12/2000 | NKG-24 | Odisha | India |
| 327 | IIb | IC282099 | 21/10/2000 | CN-8082 | Andhra Pradesh | India |
| 328 | IIb | IC283532 | 01/12/2000 | VKS/SCC-4/77 | Bihar | India |
| 329 | IIb | IC346275 | 20/03/2002 | VKS/SCC-9/46 | Bihar | India |
| 330 | IIb | IC394431 | 08/03/2003 | SMBR-344 | Assam | India |
| 331 | IIb | IC315094 | 01/03/2000 | PLM-0660 | Gujarat | India |
| 332 | IIb | IC488573 | 1994 | PLM-66, PI 363375 | Unknown | India |
| 333 | IIb | IC488884 | 1988 | PLM-642, PI 363849 | Gujarat | India |
| 334 | IIb | IC489005 | 1988 | PLM-96 | Bihar | India |
| 335 | IIb | IC553798 | 10/08/2007 |  | Uttar Pradesh | India |
| 336 | IIb | PLM-648 |  |  | Unknown | India |
| 337 | IIb | EC592173 | 2009 | VC 6141-54 |  | Taiwan |
| 338 | IIb | IC43594 | 10/07/1981 | No. 194 | Tamil Nadu | India |
| 339 | IIb | IC283411 | 27/09/2000 | PBJ/SCC-2/36 | Bihar | India |
| 340 | IIb | IC283493 | 30/11/2000 | VKS/SCC-4/38 | Bihar | India |
| 341 | IIb | IC314568 | 01/03/2000 | PLM-0333 | Uttar Pradesh | India |
| 342 | IIb | IC548266 | 01/01/1995 | Pushkara (LGG-450) UGLA 450 (PUSHKAVA) | Andhra Pradesh | India |
| 343 | IIb | IC548267 | 01/01/2006 | TM-96-2 (Trombay Pesara) TRANBOY PERAM | Andhra Pradesh | India |
| 344 | IIb | IC548268 | 01/01/2001 | Madhira-295 (MGG-295) | Andhra Pradesh | India |
| 345 | IIb | IC548275 | 01/01/1997 | ULG-37 | Telangana | India |
| 346 | IIb | IC598293 | 01/11/2013 | ML 1299 | Punjab | India |
| 347 | IIb | IC610380 | 03/12/2013 | KVSA-1784 | Arunachal Pradesh | India |
| 348 | IIb | IC626196 | 2018 | LM/14-18 | Tamil Nadu | India |
| 349 | IIb | IC507244 | IC507244 | PLM-16 | Bihar | India |
| 350 | IIb | IC607171 | 2009 |  | Unknown | India |
| 351 | IIb | IC507310 | 1988 | PLM-161 | Bihar | India |
| 352 | IIb | IC507321 |  | PLM-188, PI 363487 | Uttar Pradesh | India |
| 353 | IIb | IC507415 |  | PLM-483, PI 363725 | Haryana | India |
| 354 | IIb | IC488532 |  | PLM-514, PI 363752 | Punjab | India |
| 355 | IIb | IC488823 | 1994 | PLM-350 | Unknown | India |
| 356 | IIb | IC507517 |  | PLM-849, PI 364028 | Rajasthan | India |
| 357 | IIb | IC121177_1 |  | PLM-44, PI 363351 | Unknown | India |
| 358 | IIb | IC259534 |  |  | Unknown | India |
| 359 | IIb | IC258597 | 16/09/1999 | LB/14 | Maharashtra | India |
| 360 | IIb | IC283387 | 26/09/2000 | PBJ/SCC-2/12 | Bihar | India |
| 361 | IIb | IC267671 | 04/04/2000 | VKG13/32 | Bihar | India |
| 362 | IIb | IC417869 |  |  | Unknown | India |
| 363 | IIb | IC267672 | 09/04/2000 | VKG-13/105 | Bihar | India |
| 364 | IIb | IC424635 | 27/03/2004 | JBT-38/156 | Jharkhand | India |
| 365 | IIb | IC362577 | 2003 | 10105, Z-2/CARI/00/03 | Andaman and Nicobar Islands | India |
| 366 | IIb | IC488904 | 1988 | PLM-634 | Punjab | India |
| 367 | IIb | IC488826_1 | 1994 | PLM-33 | Unknown | India |
| 368 | IIb | IC76401 |  | M-516 | Unknown | India |
| 369 | IIb | IC364130 | 16/12/2002 | TNAU10007, Z-2/TNAUF/2002/16 | Tamil Nadu | India |
| 370 | IIb | IC76585 | 31/12/1967 | M-942 | Delhi | India |
| 371 | IIb | IC76464 | 31/12/1967 | M-640 | Delhi | India |
| 372 | IIb | IC488976 | 1988 | PLM-90 | Bihar | India |
| 373 | IIb | IC76583 | 31/12/1967 | M-940 | Delhi | India |
| 374 | IIb | IC507259 | 1988 | PLM-38 | Bihar | India |
| 375 | IIb | EC2851-3 | 1992 |  |  | Unknown |
| 376 | IIb | IC148430 | 08/09/1992 | STV-2696, NIC-15147 | Andhra Pradesh | India |
| 377 | IIb | IC305249 | 2001 |  | Unknown | India |
| 378 | IIb | IC305293 | 2001 |  | Unknown | India |
| 379 | IIb | IC343888 | 23/12/2001 | SKN-079 | Andhra Pradesh | India |
| 380 | IIb | IC472097 | 28/04/2005 | PLM-877 | Kerala | India |
| 381 | IIb | IC488506_1 | 1994 | PLM-53 | Unknown | India |
| 382 | IIb | IC488554 | 1988 | PLM-578, PI 363802 | Punjab | India |
| 383 | IIb | IC488770 | 1991 | CH-2-3 | Unknown | India |
| 384 | IIb | IC553601 | 30/07/2007 | UPM 02-17 (Pant Mung-6) | Uttarakhand | India |
| 385 | IIb | IC320902 | 24/07/2001 | VKG-18/51 | Jharkhand | India |
| 386 | IIb | IC423100 | 03/03/2004 | APGG-1 | Assam | India |
| 387 | IIb | IC597670 | 28/10/2012 | DPS/OPD-4 | West Bengal | India |
| 388 | IIb | IC488843 |  | PLM-291, PI 363582 | Unknown | India |
| 389 | IIb | IC507465 | 1987 | PLM-692 | Gujarat | India |
| 390 | IIb | IC507299 | 1988 | PLM-126, PI 363431 | Bihar | India |
| 391 | IIb | IC507293_1 | 1988 | PLM-118 | Bihar | India |
| 392 | IIb | IC436570 | 10/10/2003 | KARS-071 | Andhra Pradesh | India |
| 393 | IIb | IC436571 | 10/10/2003 | KARS-072 | Andhra Pradesh | India |
| 394 | IIb | IC436732 | 15/10/2003 | KARS-249 | Andhra Pradesh | India |
| 395 | IIb | IC488973 | 1988 | PLM-950, PI 364104 | Gujarat | India |
| 396 | IIb | IC282095 | 21/10/2000 | CN-8074 | Andhra Pradesh | India |
| 397 | IIb | EC27514 | 1987 | 54, FC no. 4413 |  | Pakistan |
| 398 | IIb | EC512789 |  | PI-376863, Ph 6920 |  | USA |
| 399 | IIb | IC39364 | 12/08/1980 | 227/4 | Rajasthan | India |
| 400 | IIb | IC488810_1 | 1994 | PLM-947, PI 364102 | Unknown | India |


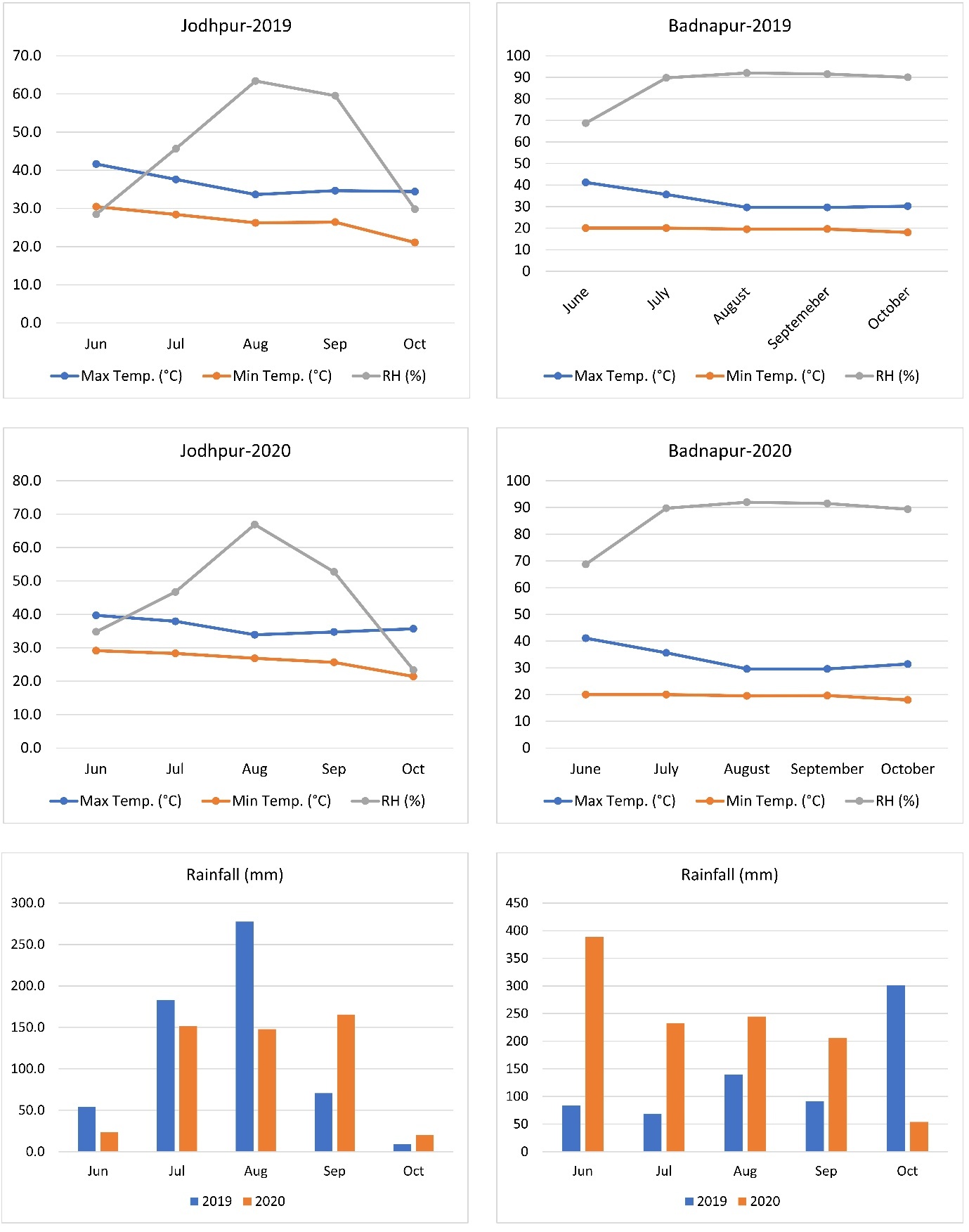


**Figure S1:** Weather data during the cropping period of the monsoon season of 2019 and 2020 at Jodhpur and Badnapur locations, where the phenotypic characterization of mungbean germplasm was done.

**Table S2:** Bartlett’s χ^2^ test to determine the homogeneity of error variances for quantitative data of the two environments for the two seasons

| **Trait** | **χ^2^ test value** | **d.f.** | **p-value** | **Significance** |
| --- | --- | --- | --- | --- |
| Terminal leaf length (cm) | 142.7 | 2 | 1.03E-31 | ** |
| Terminal leaf width (cm) | 248.3 | 2 | 1.18E-54 | ** |
| Plant height (cm) | 164.3 | 2 | 2.07E-36 | ** |
| Petiole length (cm) | 19.0 | 2 | 7.47E-05 | ** |
| Peduncle length (cm) | 106.8 | 2 | 6.58E-24 | ** |
| Pod length (cm) | 355.2 | 2 | 7.44E-78 | ** |
| No of pods per cluster | 104.3 | 2 | 2.25E-23 | ** |
| Plant stem diameter (cm) | 1744.3 | 2 | 0.00E+00 | ** |
| Number of primary branches | 172.5 | 2 | 3.46E-38 | ** |
| Number of seeds per pod | 4.7 | 1 | 2.97E-02 | * |
| Number of pods per plant | 1193.6 | 2 | 6.47E-260 | ** |
| Plant biomass (g) | 360.7 | 2 | 4.79E-79 | ** |
| Days to 50% flowering | 256.2 | 2 | 2.38E-56 | ** |
| Grain yield per plant (g) | 468.4 | 2 | 1.96E-102 | ** |
| 100-seed weight (g) | 737.3 | 2 | 7.90E-161 | ** |

**Table S3:** Analysis of variance for the phenotypic traits recorded during the season 2020 at the Jodhpur location

|  | **Source** | **Treatment (ignoring Blocks)** | **Treatment: Check** | **Treatment: Test** | **Treatment: Test vs. Check** | **Block (eliminating Treatments)** | **ⁿˢ P > 0.05; * P <= 0.05; ** P <= 0.01** |
| --- | --- | --- | --- | --- | --- | --- | --- |
| **Terminal leaf length (cm)** | Df | 3907 | 4 | 3902 | 1 | 50 | 455 |
|  | Mean.Sq | 1.30 ** | 39.48 ** | 1.25 ** | 33.68 ** | 2.50 | 0.99 |
| **Terminal leaf width (cm)** | Df | 3907 | 4 | 3902 | 1 | 50 | 455.00 |
|  | Mean.Sq | 1.22 ** | 49.36 ** | 1.16 ** | 44.26 ** | 2.06 | 0.86 |
| **Plant height (cm)** | Df | 3907 | 4 | 3902 | 1 | 50 | 455.00 |
|  | Mean.Sq | 218.18 ** | 3839.84 ** | 198.89 ** | 60985.07 ** | 102.21 | 48.33 |
| **Petiole length (cm)** | Df | 3907 | 4 | 3902 | 1 | 50 | 455.00 |
|  | Mean.Sq | 3.58 ** | 43.02 ** | 3.54 ** | 0.23 ⁿˢ | 7.50 | 2.26 |
| **Peduncle length (cm)** | Df | 3907 | 4 | 3902 | 1 | 50 | 455.00 |
|  | Mean.Sq | 18.93 ** | 186.80 ** | 18.07 ** | 2680.06 ** | 18.82 | 8.08 |
| **Pod length (cm)** | Df | 3907 | 4 | 3902 | 1 | 50 | 455.00 |
|  | Mean.Sq | 1.67 ** | 142.84 ** | 1.50 ** | 116.53 ** | 0.45 | 0.42 |
| **No of pods per cluster** | Df | 3907 | 4 | 3902 | 1 | 50 | 455.00 |
|  | Mean.Sq | 1.73 ** | 18.80 ** | 1.64 ** | 274.07 ** | 1.66 | 0.76 |
| **Plant stem diameter (mm)** | Df | 3907 | 4 | 3902 | 1 | 50 | 455.00 |
|  | Mean.Sq | 4.99 ** | 28.37 ** | 4.85 ** | 448.96 ** | 2.94 | 1.86 |
| **Number of primary branches** | Df | 3907 | 4 | 3902 | 1 | 50 | 455.00 |
|  | Mean.Sq | 1.38 ** | 16.69 ** | 1.32 ** | 198.88 ** | 1.25 | 0.66 |
| **Number of seeds per pod** | Df | 3907 | 4 | 3902 | 1 | 50 | 455.00 |
|  | Mean.Sq | 1.08 ** | 15.38 ** | 1.06 ** | 0.45 ⁿˢ | 1.22 | 0.55 |
| **Number of pods per plant** | Df | 3907 | 4 | 3902 | 1 | 50 | 455.00 |
|  | Mean.Sq | 587.64 ** | 9972.05 ** | 568.79 ** | 36594.41 ** | 831.67 | 384.42 |
| **Plant biomass (g)** | Df | 3907 | 4 | 3902 | 1 | 50 | 455.00 |
|  | Mean.Sq | 506.24 ** | 5048.49 ** | 484.96 ** | 65340.63 ** | 222.85 | 146.65 |
| **Days to 50% flowering** | Df | 3907 | 4 | 3902 | 1 | 50 | 455.00 |
|  | Mean.Sq | 86.84 ** | 822.04 ** | 77.21 ** | 34745.48 ** | 29.36 | 11.27 |
| **Days to 80% maturity** | Df | 3907 | 4 | 3902 | 1 | 50 | 455.00 |
|  | Mean.Sq | 63.21 ** | 326.63 ** | 58.07 ** | 19061.56 ** | 91.86 | 22.01 |
| **Grain yield per plant (g)** | Df | 3907 | 4 | 3902 | 1 | 50 | 455.00 |
|  | Mean.Sq | 48.87 ** | 69.17 ⁿˢ | 46.01 ** | 11110.98 ** | 77.72 | 38.47 |
| **100-seed weight (g)** | Df | 3907 | 4 | 3902 | 1 | 50 | 455.00 |
|  | Mean.Sq | 1.06 ** | 38.87 ** | 1.00 ** | 108.18 ** | 0.38 | 0.26 |
| **Seed area (mm^2^)** | Df | 3796 | 4 | 3791 | 1 | 50 | 454.00 |
|  | Mean.Sq | 9.57 ** | 361.75 ** | 9.08 ** | 453.78 ** | 2.84 | 2.07 |
| **Seed length (mm)** | Df | 3796 | 4 | 3791 | 1 | 50 | 454.00 |
|  | Mean.Sq | 0.27 ** | 11.55 ** | 0.26 ** | 17.69 ** | 0.09 | 0.06 |
| **Seed breadth (mm)** | Df | 3796 | 4 | 3791 | 1 | 50 | 454.00 |
|  | Mean.Sq | 0.14 ** | 3.29 ** | 0.13 ** | 4.84 ** | 0.08 | 0.04 |
| **Seed roundness** | Df | 3796 | 4 | 3791 | 1 | 50 | 454.00 |
|  | Mean.Sq | 0.00 ** | 0.02 ** | 0.00 ** | 0.02 ** | 0.00 | 0.00 |
| **Pod weight (g)** | Df | 3907 | 4 | 3902 | 1 | 50 | 455.00 |
|  | Mean.Sq | 113.14 ** | 251.65 * | 107.10 * | 23123.31 ** | 215.83 | 91.11 |





**Figure S2:** Bar chart frequency distribution of quantitative phenotypic parameters recorded on entire collections (EC) of mungbean at Jodhpur location (2019 and 2020) and Badnapur (2019).


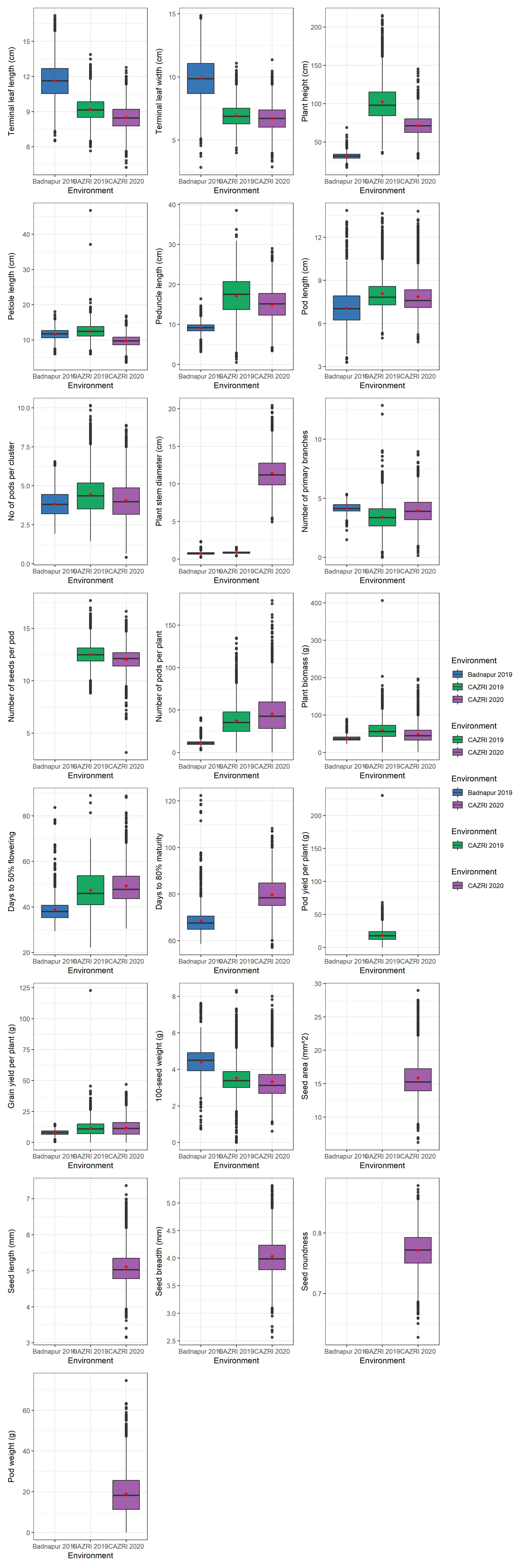


**Figure S3:** Box-plot frequency distribution of quantitative phenotypic parameters recorded on entire collections (EC) of mungbean at Jodhpur location (2019 and 2020) and Badnapur (2019).


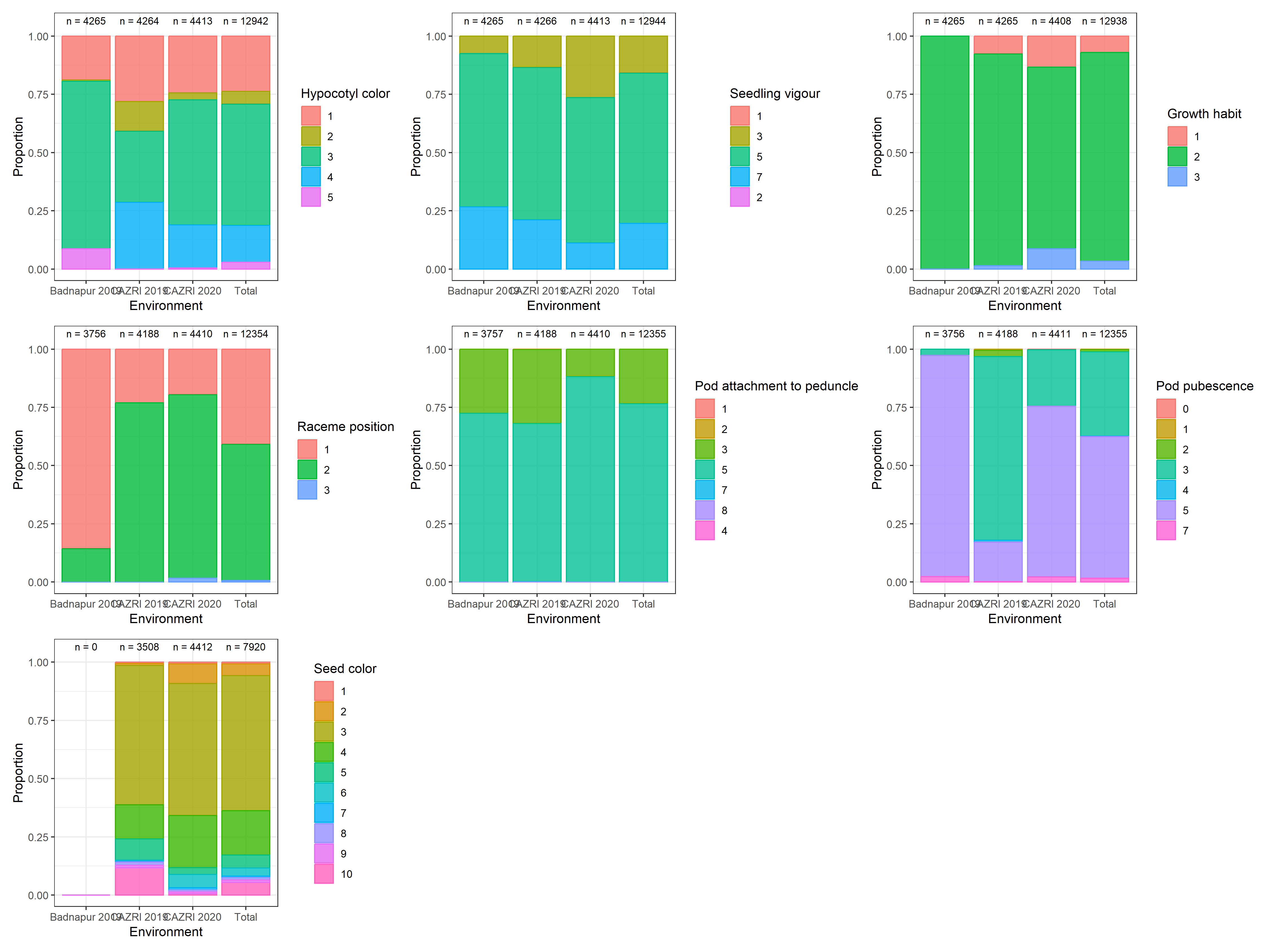


**Figure S4:** Bar chart frequency distribution of qualitative phenotypic parameters recorded on entire collections (EC) of mungbean at Jodhpur location (2019 and 2020) and Badnapur (2019).


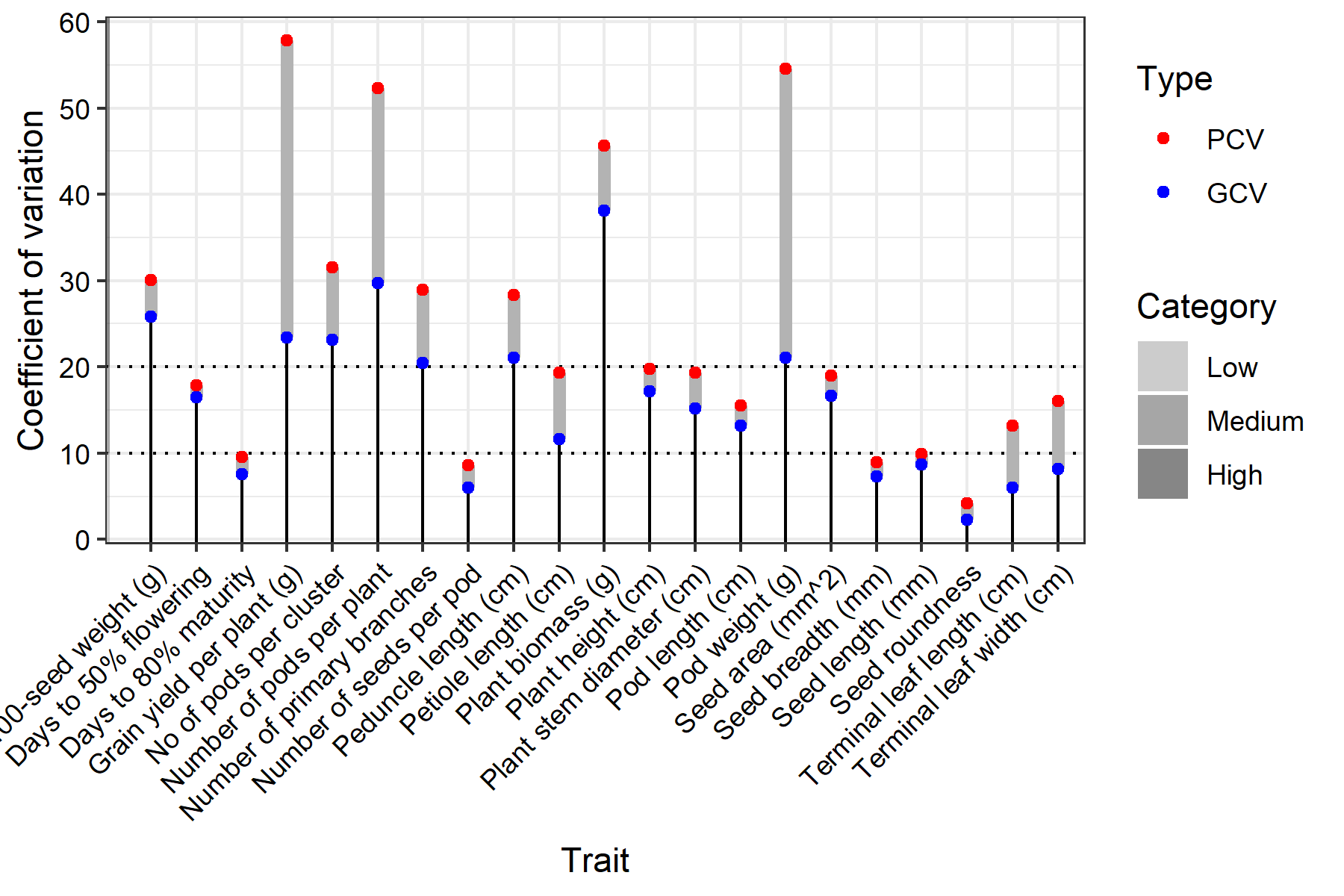


**Figure S5:** Gross value-added plot highlighting the GCV and PCV for quantitative phenotypic traits in the entire collection (EC) of mungbean germplasm


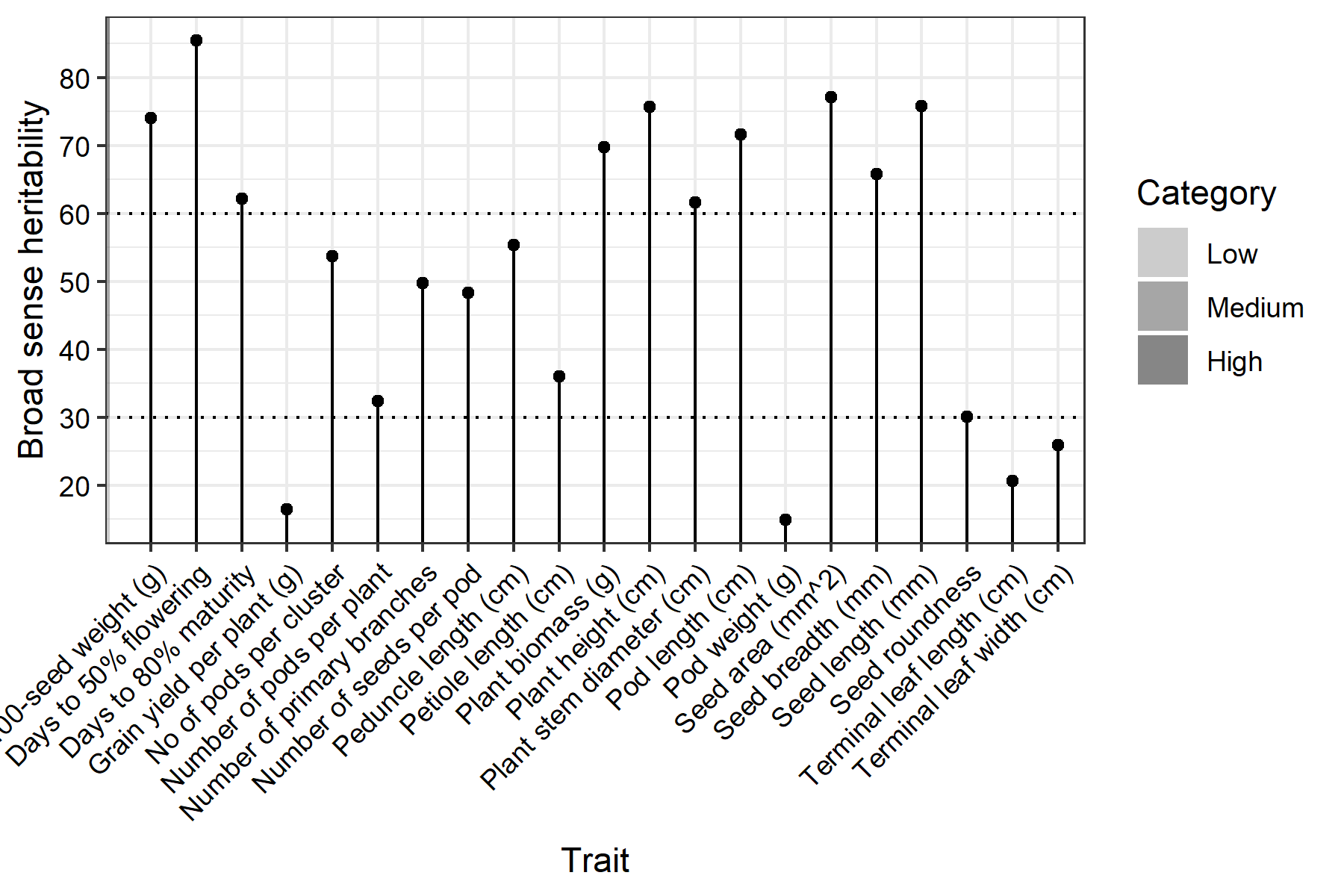


**Figure S6:** Gross value-added plot highlighting the broad-sense heritability (h^2^) for quantitative phenotypic traits in the entire collection (EC) of mungbean germplasm

**Table S4:** List of promising accessions identified based on the critical difference (CD at 5% significance level) for traits of breeders’ importance

| **Trait** | **Range** | **Promising accessions** | **Best check variety** | **CD at 5%** |
| --- | --- | --- | --- | --- |
| Days to 80% maturity | 59.0-110 | ≤ 61 days: IC113983, IC119026, S-9 | MH421 (71.41) | 10.20 |
| Pod length (cm) | 4.6-13.8 | ≥ 12.7 cm: IC626176, EC396410, EC396133, EC396141, IC332332, EC396402, IC331228, C607183, EC396113, IC326721, EC398885 | SML668 (10.32) | 1.42 |
| Number of pods/cluster | 1.0-9.0 | ≥ 8: IC436570, IC2029, IC385717, IC282155, IC148541, IC24782, IC118954, IC315094, IC148494 | PDM139 (5.36) | 1.90 |
| Plant stem diameter (mm) | 5.2-19.7 | ≥ 18.0 mm: IC39493, IC121200, IC39459, IC39448, IC52046, IC488603, IC436783, IC39415, IC488781, IC489098, IC8972-1 | IPM02-3 (10.95) | 2.97 |
| Number of seeds/pod | 6.3-16.3 | ≥ 15: EC528091, IC436633, IC118956, IC273267, IC682, IC338852, IC507291, IC436726, IC610282, IC283493, IC267672, PLM707, IC336750, IC148516 | SML668 (12.55) | 1.61 |
| Plant biomass/plant (g) | 8.0-194 | ≥ 130 g: IC121315, IC39493, IC472098, IC39491, IC472099, IC11438-4, IC395516, IC472084 | PM02-3 (46.36) | 26.32 |
| Grain yield/plant (g) | 1.0-46.4 | ≥ 32 g: IC470530, IC395518, IC548369, EC245967, IC10483-3, IC73359, EC314302, IC24782, IC436571, EC245966-1 | IPM2-14 (17.58) | 13.48 |
| 100 seed weight (g) | 1.17-8.0 | ≥ 6.84 g: EC251967-1, EC398917, EC592176, EC590222, IC607146, EC396104, EC398884, EC396409, EC272458, IC607185, EC396122 | SML668 (4.79) | 1.11 |


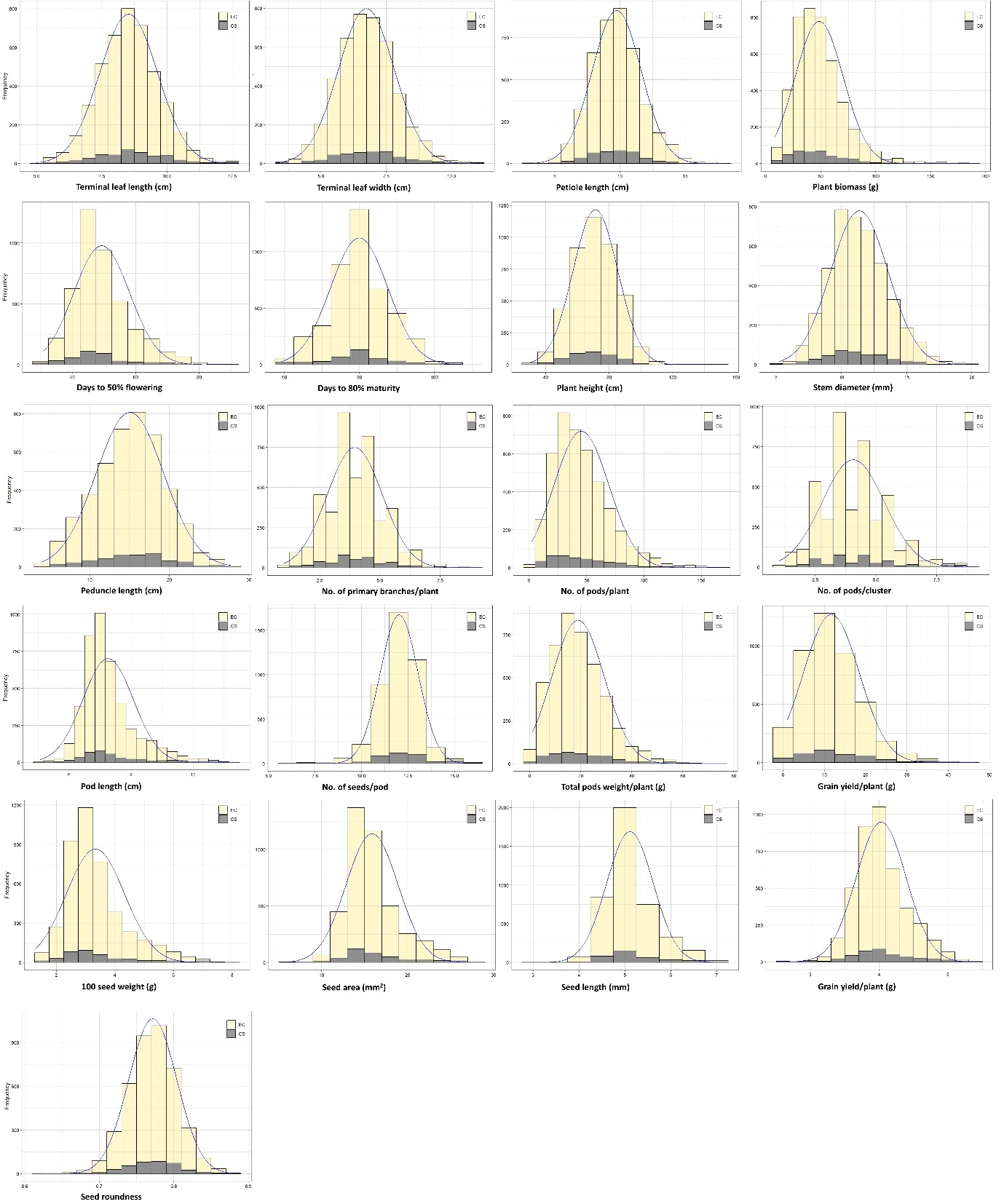


**Figure S7:** Frequency distribution charts showing the comparative distribution of the phenotypic diversity in the entire collection (yellow colour bars) and the core collection (grey colour within the yellow colour bars) recorded for the season 2020 at Jodhpur.


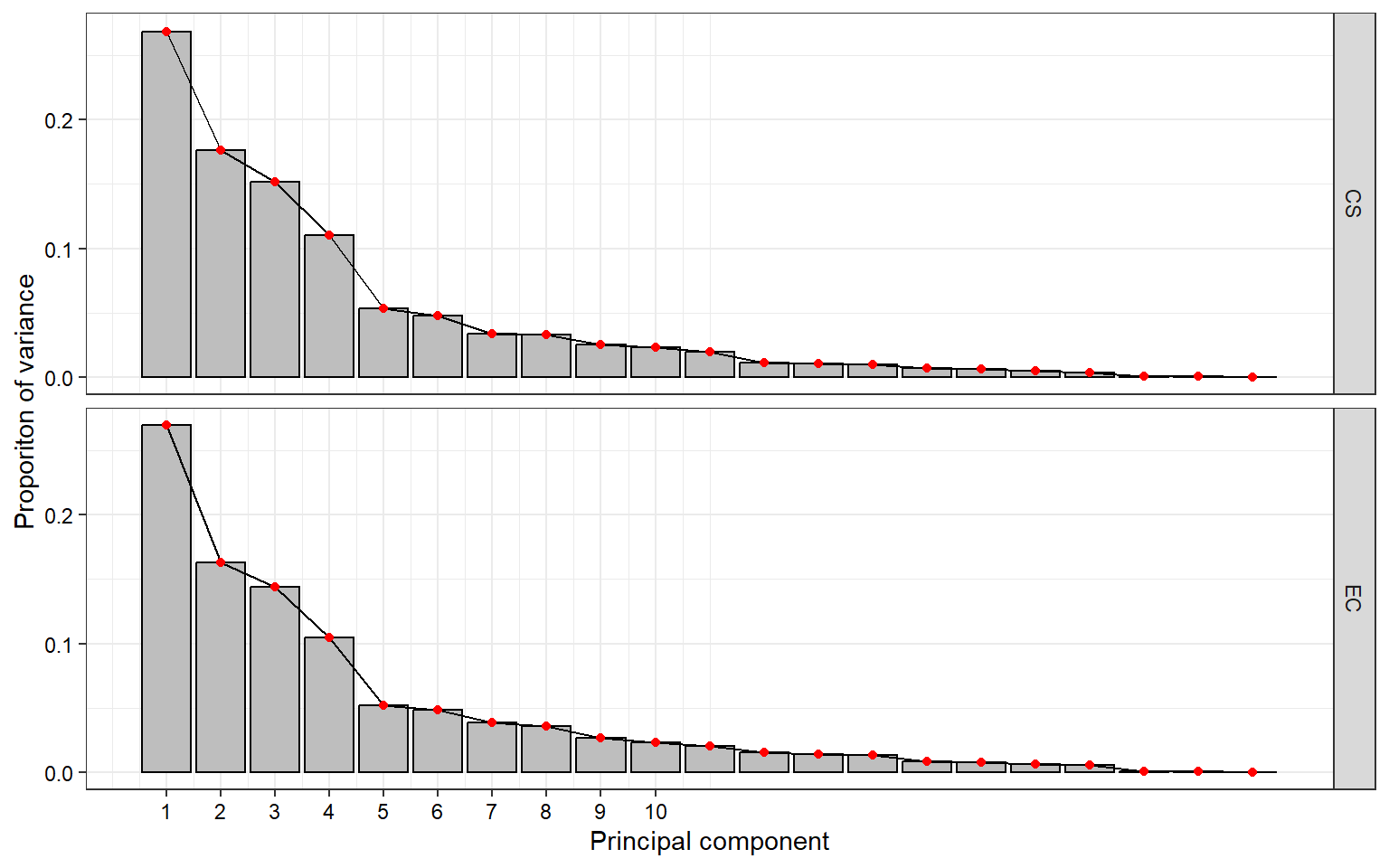


**Figure S8:** Scree chart, a graphical representation of the proportion of variance explained by principal components (PCs). The elbow point (point of inflection) indicates that from PC5 onward, the rate of variance decrease for the rest of the PCs is flattened, indicating the largest proportion of variance was governed by the first four PCs. The chart indicates a similar pattern of variance distribution over the PCs in the entire collection (EC) as well as the core collection (CS).

**
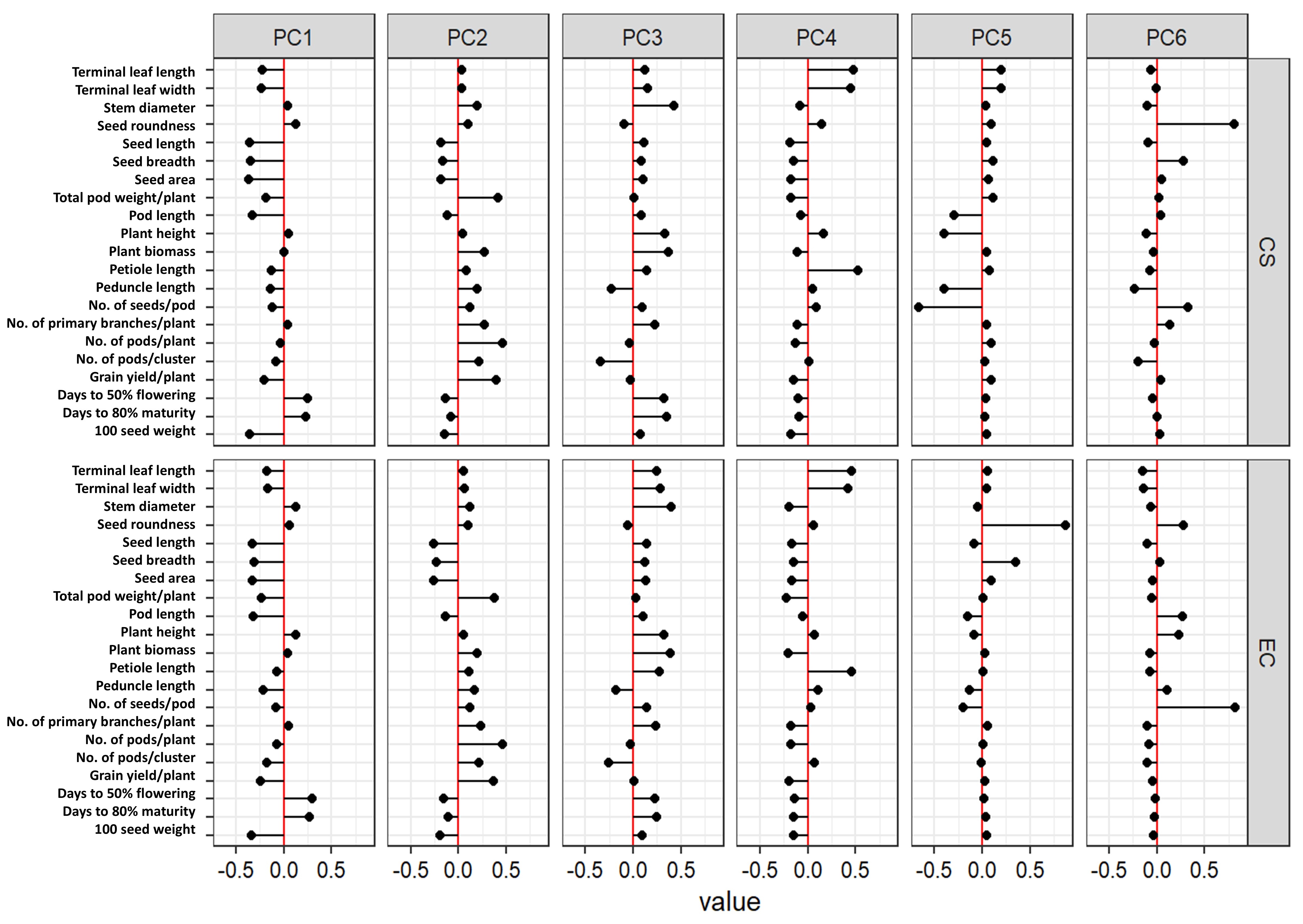
**

**Figure S9:** Visualization of the first six PCs and loading values (bar lines) and their directions for phenotypic traits in the entire collections (EC) as well as the core collection (CS). The first four PCs revealed a similar pattern between EC and CS for the direction and magnitude of contribution of loading values for each trait. The PC 5 and PC 6 show significant deviations between EC and CS. This indicates that the first core collections have captured a major proportion of the variation of the entire mungbean collection. The minor deviation in variance is primarily contributed by the traits such as plant height, peduncle length, no. of seeds/pod, and seed roundness.
